# *Prevotella copri* alleviates diarrhea in weaning piglets through gut microbiota modulation and arachidonic acid–AHR–NRF2 pathway activation

**DOI:** 10.1186/s40104-025-01273-y

**Published:** 2025-11-20

**Authors:** Cong Lan, Wen Ren, Aimin Wu, Bing Yu, Jun He, Yuheng Luo, Daiwen Chen

**Affiliations:** Key Laboratory for Animal Disease-Resistance Nutrition of Ministry of Education of China, Key Laboratory for Animal Disease-Resistance Nutrition and Feed of Ministry of Agriculture of China, Key Laboratory of Animal Disease-Resistant Nutrition of Sichuan Province, Engineering Research Center of Animal Disease-Resistance Nutrition Biotechnology of Ministry of Education of China, Animal Nutrition Institute, Sichuan Agricultural University, Chengdu, China

**Keywords:** Arachidonic acid metabolism, Diarrhea, Microbial ecological network, *Prevotella copri*

## Abstract

**Background:**

Diarrhea remains a major health concern in both young animals and humans. *Prevotella* spp., a dominant commensal genus in the healthy porcine gut, becomes increasingly abundant following weaning, suggesting a potential role during this critical transitional period. However, its involvement in post-weaning diarrhea remains poorly understood. Here, we aim to elucidate the role and underlying mechanisms of *Prevotella* in alleviating diarrhea in weaned piglets.

**Results:**

To model unsanitary housing conditions, piglets were housed in uncleaned pens containing residual fecal matter from previous occupants and exposed to cold stress by maintaining the ambient temperature at 19 °C, below the optimal 28 °C. Under these conditions, piglets were orally administered either a blank medium (CON, *n* = 10 × 2) or *Prevotella copri* at 1 × 10^8^ CFU (Pc, *n* = 10 × 2) on d 1, 3, and 5. After 28 d, cold stress induced a diarrhea incidence of 33.45% in the CON group, while *P. copri* supplementation significantly reduced the diarrhea rate to 19.73%. Treatment with *P. copri* markedly improved intestinal morphology in the small intestine, decreased serum levels of lipopolysaccharide (LPS) and intestinal fatty acid-binding protein (i-FABP), and enhanced total antioxidant capacity (T-AOC) and catalase (CAT) activity. Quantitative PCR and 16S rRNA gene sequencing revealed that *P. copri* significantly increased the colonic abundance of *Prevotella*, reshaping both the composition and functional profile of the gut microbiota. Moreover, *P. copri* enhanced the modularity and robustness of microbial ecological networks. Untargeted metabolomic profiling of colonic contents revealed a significant enrichment of metabolites involved in the arachidonic acid pathway following *P. copri* supplementation. In parallel, untargeted metabolomics of *P. copri* culture supernatants identified differential metabolic pathways including metabolic pathways, biosynthesis of secondary metabolites, and biosynthesis of antibiotics. In vitro assays demonstrated that *P. copri*-derived metabolites inhibited the growth of three common porcine intestinal pathogens. Furthermore, both *P. copri* metabolites and arachidonic acid enhanced intestinal barrier integrity and suppressed TNF-α-induced inflammation and apoptosis in Caco-2 cells through activation of the AHR–Nrf2 signaling pathway.

**Conclusions:**

These findings highlight the role of *P. copri* in maintaining gut homeostasis and provide new insights into microbiota-based interventions for early-life intestinal disorders.

**Supplementary Information:**

The online version contains supplementary material available at 10.1186/s40104-025-01273-y.

## Background

Diarrhea presents a significant challenge for young animals, including both infants and neonates of livestock species. Notably, diarrhea is recognized as a major cause of malnutrition in children under the age of five [1]. In livestock production, weaning stress often leads to severe diarrhea, particularly in piglets [2]. Given the remarkable similarities between pigs and humans in terms of metabolism and genetic attributes, pigs serve as an attractive and reliable biomedical model for studying human diseases, including diarrhea in a human-relevant context. Current research has demonstrated that weaning stress, encompassing nutritional, physiological, and psychological factors, induces alterations in intestinal morphology, physiological function, and the composition of the gut microbiota in piglets [3–5]. Among these, gut microbiota dysbiosis is considered a major mechanism contributing to the development of diarrhea [6]. 

The gut microbiota of piglets undergoes marked changes around the weaning period, characterized by reduced β-diversity and shifts in species composition [7]. Our meta-analysis of the dynamic changes in gut microbiota across different ages and growth stages in pigs revealed that, prior to weaning, the gut microbiota is primarily dominated by *Bacteroides*, *Escherichia*, *Clostridium*, *Lactobacillus*, *Fusobacterium*, and *Prevotella* [8]. Post-weaning, as piglets age, the microbial community undergoes a transition marked by *Prevotella* emerging as a core genus. Notably, once piglets begin consuming solid feed, the relative abundance of *Prevotella* significantly increases from 12.93% on the day of weaning to 57.24% by 7 d post-weaning [9, 10]. A *Prevotella*-dominated enterotype has been positively associated with animal growth performance, including feed intake [11], feed efficiency [12], and weight gain [13]. However, these findings have predominantly focused on growing-finishing pigs, with relatively limited research on weaned piglets. *Prevotella* is likely closely linked to post-weaning diarrhea (PWD) in piglets [14], yet further validation through integrative multi-omics approaches remains lacking. *Prevotella copri* is a representative species within the *Prevotella* genus and has been extensively studied in both humans and animals [15, 16]. It is specifically adapted to the host intestinal environment and is reported to be the most prevalent *Prevotella* member in the porcine gut microbiota [17]. Given its dominance and host specificity, *P. copri* was selected as a model strain for further investigation in this study.

Based on our previous findings and the results from other studies, we hypothesize that *Prevotella* may represent a key beneficial symbiont involved in modulating gut microbial homeostasis [18] and alleviating post-weaning diarrhea in piglets [8]. Therefore, in this study, we first successfully established a stress-induced diarrhea model in weaned piglets and investigated the effects of *P. copri* administration on growth performance and gut health-related phenotypes. Subsequently, by analyzing the microbial composition, microbial ecological networks, and metabolite profiles in colonic digesta, we identified that *P. copri* may influence gut health in diarrheic piglets through modulation of the gut microbiota and arachidonic acid metabolism. Finally, bacterial co-culture and in vitro cellular assays were conducted to elucidate the potential mechanisms by which *P. copri* alleviates epithelial damage in the host intestine. Collectively, our findings provide valuable new insights into targeting the gut microbiota as a therapeutic strategy against diarrhea in young mammals, including infants.

## Methods

### Preparation of bacteria

The reference strain of *Prevotella copri* (*P. copri* DSMZ 18205, Guangdong Microbial Culture Collection Center, China) was purchased in powder form. The culture medium formulation was based on our previous studies [19].

### Animal model construction, grouping, and feeding management

A total of 40 crossbred (Duroc × Landrace × Yorkshire) piglets (21 days old) with similar body weight and sex ratio were randomly divided into two groups: control (CON) and *P. copri*-treated (Pc), with 10 replicates per group and two piglets per replicate. The trial lasted 28 d. On d 1, 3, and 5, piglets in group Pc received 10 mL of *P. copri* culture (10^8^ CFU/mL) by oral gavage using a medical nasal feeding tube, while those in the CON group were administered an equal volume of sterile culture medium. The dosage of *P. copri* used in this study was based on previously reported protocols [16]. All animals were fed a corn-soybean meal-based pelleted diet (Table S1) formulated to meet NRC (2012) nutritional recommendations [20].

The experiment was conducted in a weanling pig barn at DSM (China) Animal Nutrition R&D Center. A standardized stress model was applied based on Dou et al. [14], including: (1) uncleaned pens covered with fecal matter from previous piglets to simulate unsanitary housing, and (2) maintaining barn temperature at 19 °C (below the optimal 28 °C) to induce cold stress. The study was conducted during winter to enhance consistency of stress exposure. Water and feed were provided ad libitum. No antibiotics or probiotics were used during the trial, and daily feed intake and health status were closely monitored.

### Growth performance and diarrhea monitoring

Piglet body weights were measured after overnight fasting on d 1, 14, and 28. Fecal scores were recorded daily throughout the trial according to predefined criteria (Table S2), and diarrhea incidence was calculated as: Diarrhea rate (%) = (number of diarrhea cases)/(total piglets × trial days) × 100. 

### Sample collection

On d 28, eight replicates per group (16 piglets in total, sex-balanced, near-average body weight) were selected for sampling. After overnight fasting, 10 mL of blood was collected via the anterior vena cava, allowed to clot at room temperature, then centrifuged (3,000 × *g*, 15 min, 4 °C) to isolate serum. Subsequently, pigs were euthanized and intestinal segments (duodenum, jejunum, ileum, colon) were collected under sterile conditions. Chyme was preserved in sterile cryovials, while each segment was rinsed with cold PBS and blotted dry. A 5 cm section of small intestine was fixed in 4% paraformaldehyde for histological analysis. Remaining intestinal tissue was longitudinally opened, and mucosa was scraped with a sterile slide and stored.

### Histological analysis of intestinal morphology

Segments of the duodenum, jejunum, and ileum fixed in 4% paraformaldehyde were processed through standard dehydration, clearing, and paraffin embedding procedures. Tissue sections were cut at a thickness of 5 μm and stained using the Periodic acid-Schiff (PAS) method. After mounting, the slides were examined under a light microscope. Ten well-oriented fields with intact villi were randomly selected per section to assess mucosal thickness, villus length, villus width, crypt depth, and villus spacing.

### Microbial composition analysis and *Prevotella* quantification in colonic digesta

On d 28, colonic digesta samples were collected for 16S rRNA amplicon sequencing to assess microbial diversity. For 16S rRNA sequencing, 10 biological replicates were analyzed per group, with each replicate representing one piglet (*n* = 10 piglets per group). Genomic DNA was extracted using the E.Z.N.A.^®^ Stool DNA Kit (Omega Bio-Tek, USA). The V3–V4 region of 16S rRNA was amplified using primers 341F and 785R, and PCR products were purified, pooled, and sequenced on the Illumina MiSeq platform (USA). Sequencing data were quality filtered using fastp and FLASH by trimming low-quality bases (Q < 20) with a sliding window, discarding reads < 50 bp or with > 5 ambiguous bases. Paired-end reads were merged (≥ 10 bp overlap, ≤ 0.2 mismatch ratio) and demultiplexed with no barcode mismatches and up to two primer mismatches. Sequencing data were processed with Mothur v1.44.0 for quality control and OTU clustering. UCHIME v4.2 was used to remove chimeric sequences. OTUs were taxonomically assigned using the Ribosomal Database Project (RDP v2.6). To minimize sequencing bias, all samples were rarefied to the same sequencing depth (i.e., normalized to the minimum read count across samples). Low-abundance OTUs were filtered out (threshold < 0.01% of total reads). Alpha and beta diversity analyses were performed using the *vegan* and *phyloseq* packages in R (v3.6.3). Differential abundance analysis was conducted using Welch’s *t*-test (two-tailed), and confidence intervals were estimated by Welch’s inverted method at the 95% confidence level. Microbial diversity analysis was performed using QIIME2 and R (version 3.3.1). Alpha diversity was evaluated using the Shannon and Simpson indices to estimate microbial richness and evenness within samples. These metrics were calculated with QIIME2. Beta diversity was assessed using both supervised and unsupervised approaches. Principal Coordinates Analysis (PCoA) based on Bray–Curtis dissimilarity was conducted in R (version 3.3.1) to visualize differences in microbial community structure across groups. Statistical significance of these differences was tested using Permutational Multivariate Analysis of Variance (PERMANOVA), also performed in R (version 3.3.1) with 999 permutations. In addition, Partial Least Squares Discriminant Analysis (PLS-DA) was conducted using the plsda function in the *mixOmics* package (R version 3.3.1) to identify patterns of group separation based on microbial composition and for visualization of discriminative features.

Correlation analyses were conducted using Spearman’s rank correlation method, and statistical analyses and visualizations were carried out using the OmicStudio platform (https://www.omicstudio.cn/tool/62). A *P* value < 0.05 was considered statistically significant. KEGG pathway functions were predicted using PICRUSt2, and differences between groups were assessed using the Wilcoxon rank-sum test, with two-tailed testing and a 95% confidence interval. A *P* value < 0.05 was considered statistically significant. Microbial ecological networks were constructed using the Molecular Ecological Networks (MENs) based on the Random Matrix Theory (RMT) algorithm available on the iNAP2 platform, following the platform’s standard parameter settings [21].

Fecal samples were collected from piglets at the beginning and end of the experiment. Absolute quantification of *Prevotella* in feces was performed using real-time PCR with genus-specific primers (forward: 5'-CACCAAGGCGACGATCA-3'; reverse: 5'-GGATAACGCCTGGACCT-3') [18]. Standard curves were generated using serial dilutions of plasmids containing the target *Prevotella* 16S rRNA gene fragment. The bacterial load was calculated and expressed as log₁₀ copies of *Prevotella* per gram of feces.

### Metabolomic analysis of *P. copri* culture supernatant and colonic digesta

*P. copri* was anaerobically cultured in sterile bottles containing blank medium at 37 °C for 48 h. Culture supernatants were collected from two groups: uninoculated medium (CON, *n* = 3) and medium inoculated with *P. copri* (Pc, *n* = 3), with sampling aligned to the optimal 48 h growth phase. All supernatants were stored at −80 °C for subsequent metabolomic analysis. Samples were thawed at 4 °C and 100 μL was transferred into 96-well plates. Extraction solvent containing internal standards (300 μL) was added, and plates were shaken (1 min), incubated at −20 °C (2 h), and centrifuged at 4,000 r/min (30 min, 4 °C). Supernatants (300 μL) were transferred to new plates and dried under vacuum. Residues were reconstituted in 150 μL methanol:water (1:1, v/v), vortexed (3 min), and centrifuged again. A pooled quality control (QC) sample was prepared by combining 10 μL from each well. Remaining samples (40 μL) were divided into plates for positive/negative ion detection and backup.

Colonic digesta (~80 mg per sample) from four replicates (two pigs per replicate, matched by average body weight) were processed in parallel. Bacterial suspensions were filtered (0.2 μm) to remove cells, and the filtrates were aliquoted (1 mL) for metabolite profiling.

Untargeted metabolomics was performed by Shanghai Applied Proteome Technology Co., Ltd. using UPLC-Q-TOF/MS (Waters) [22]. Raw data were converted to mzXML format via ProteoWizard and processed with XCMS for peak detection, alignment, and quantification. Metabolites were annotated based on accurate mass (tolerance < 25 ppm), MS/MS spectra, and reference databases from the service provider. Data analysis included multivariate (Partial least squares discriminant analysis, PLS-DA with Pareto scaling) and univariate (Student’s *t*-test) methods. Metabolites with Variable Importance in Projection (VIP) > 1 and *P* < 0.05 were considered significantly different. Progenesis QI (v2.0) was used for original data processing. Identified metabolites were annotated and mapped to pathways using HMDB and KEGG databases (http://www.kegg.jp/). Correlation analyses (*r* > 0.50 or < −0.50, *P* < 0.05) and visualization were conducted in R (v3.6.3) and via Cytoscape 3.10.0. Enrichment analysis was performed using the MetaboAnalyst 6.0 platform[23].

### Extraction of bacterial metabolites

Ethyl acetate was chosen because it efficiently extracts extracellular low-to-moderate polarity metabolites from bacterial culture supernatants and has been widely used in microbial metabolomics protocols. Metabolites from *P. copri* were extracted following a previously reported protocol [24]. Briefly, culture supernatants were obtained from both *P. copri* cultures and uninoculated blank medium (used as the control) by centrifugation at 6,000 r/min for 5 min after thawing at 4 °C. Equal volumes of ethyl acetate were added to the supernatant and mixed for 5 min to extract metabolites. After centrifugation at 1,000 r/min for 10 min, the organic phase containing metabolites was collected and evaporated under nitrogen. The dried extract was redissolved in 1 mL of culture medium, filtered through a 0.22-μm membrane to remove residual bacteria, and stored at −20 °C for subsequent assays.

### In vitro co-culture with pathogenic bacteria

Three representative pig-derived intestinal pathogens were used: Enterotoxigenic *Escherichia coli* (ETEC, College of Veterinary Medicine, Sichuan Agricultural University), *Salmonella choleraesuis* (ATCC 14028, donated by Sichuan University), and *Clostridium perfringens* (ATCC 13124, dsm-firmenich Research Center of Animal Nutrition and Health). After inoculation, all strains were cultured anaerobically for 24 h to prepare bacterial suspensions.

Each pathogen was cultured under anaerobic conditions with three treatments: LB (pathogen + blank medium), LB + ethyl acetate (CON + blank extract), and LB + *P. copri* (CON + *P. copri* extract). The LB and LB + ethyl acetate groups had three replicates each, and the LB + *P. copri* group had six replicates. Cultures were incubated at 37 °C, and bacterial growth was monitored by measuring OD_600_ every 2 h. The growth curve was constructed by plotting the derivative of ln (OD_600_) over time. Growth parameters, including the maximal growth rate (min⁻^1^) and lag phase duration, were calculated using Origin software.

### Assessment of membrane permeability in pathogenic bacteria

The membrane integrity of enterotoxigenic *Escherichia coli* (ETEC) was assessed via propidium iodide (PI), a fluorescent dye that exclusively permeates cells with impaired membrane integrity [25]. Briefly, ETEC cells cultured to the stationary growth phase were harvested via centrifugation at 300 r/min for 5 min and resuspended in sterile phosphate-buffered saline (PBS) to their original volume. PI (Sigma-Aldrich, USA) was introduced into the bacterial suspension at a final concentration of 60 μmol/L, and the resultant mixture was subsequently transferred to black 96-well plates (Greiner Bio-One, Corning, USA) at a volume of 100 μL per well. Thereafter, the wells were subjected to four distinct treatments: the control (CON) group (PBS, 1:1 dilution, *n* = 3), the cetyltrimethylammonium bromide (CTAB) group (final concentration: 300 mmol/L, *n* = 3), the polymyxin group (5 × minimum inhibitory concentration (MIC) of polymyxin, *n* = 3), and the *P. copri* extract group (*P. copri* metabolite extract, 1:1 dilution, *n* = 3). CTAB and polymyxin were employed as positive controls for membrane disruption: CTAB, a chemical compound, is known to induce pore formation in microbial membranes, whereas polymyxin is a membrane-targeting antibiotic that exerts bactericidal effects by inducing membrane permeabilization. Both agents facilitate the entry of PI into the cytoplasm, where it binds to nucleic acids (DNA and RNA), thereby generating a fluorescent signal. The treated plates were then incubated under anaerobic conditions at 37 °C for 30, 60, and 120 min, respectively. Fluorescence intensity was quantified using a microplate reader (Synergy Mx, BioTek, USA) at excitation and emission wavelengths of 530 nm and 590 nm, respectively. The fluorescence intensity of the CTAB group was designated as the reference for 100% membrane permeability, and the relative membrane permeability of the other treatment groups was calculated accordingly. All experiments were conducted in triplicate.

### Effect of *P. copri* metabolites and arachidonic acid on TNF-α-stimulated Caco-2 cells

Caco-2 cells (National Collection of Authenticated Cell Cultures, China) were cultured in high-glucose DMEM supplemented with GlutaMAX™, pyruvate, 10% fetal bovine serum, and 1% penicillin–streptomycin (all from Gibco, USA). Medium was replaced every 2–3 d, and cells were passaged at 80%–90% confluency using 0.25% trypsin (Thermo Fisher Scientific, USA) without EDTA. Cells were subculture and used according to the experimental schedule. A two-factor experimental design was employed to assess the regulatory effects of *P. copri* metabolites and arachidonic acid (ARA, Sigma-Aldrich, Cat: A3611) on TNF-α-induced inflammation in Caco-2 cells. Cells were pre-treated with either bacterial metabolites (1×, referring to a concentration equivalent to that present in the original bacterial culture supernatant prior to extraction, the reconstituted metabolites were diluted to match the original volume of the culture medium used for extraction) or 50 μmol/L ARA for 2 h, followed by stimulation with 150 ng/mL TNF-α (MedChemEcpress, USA) for 24 h. The experimental groups included: (1) CON: PBS for 26 h; (2) TNF-α: PBS for 2 h, then TNF-α for 24 h; (3) Pc or ARA: *P. copri* metabolites or ARA for 2 h, then PBS for 24 h; and (4) Pc or ARA + TNF-α: pre-treatment with *P. copri* metabolites or ARA followed by TNF-α. Each condition was tested in quadruplicate, and the experiment was repeated independently three times. Culture supernatants were collected for ELISA to quantify inflammatory cytokines.

### Apoptosis analysis by flow cytometry

Caco-2 cells were collected following treatment using trypsin (without EDTA), then centrifuged at 300 × g for 5 min to remove the supernatant. The cells were subsequently stained using an Annexin V–FITC/PI apoptosis detection kit (Beyotime Biotechnology, Shanghai, China), according to the manufacturer’s instructions. Flow cytometric analysis was conducted to quantify apoptotic cells, and the data were analyzed using FlowJo software (version 8.7; Tree Star Inc., Ashland, OR, USA).

### RNA isolation and qPCR

To evaluate gene expression changes in Caco-2 cells and tissue samples following treatment, total RNA was extracted using TRIzol reagent (Takara, Japan) according to the manufacturer’s instructions. cDNA synthesis was performed with the HiScript III RT kit (Vazyme, China). Quantitative PCR was conducted using SYBR Green Master Mix (Vazyme) and gene-specific primers (Table S3). *ACTN* and *GAPDH* served as internal controls. Relative gene expression was calculated using the 2^–ΔΔCt^ method. *P*-value < 0.05 was considered statistically significant.

### Serum biochemical analysis

Serum levels of lipopolysaccharide (LPS), intestinal fatty acid binding protein (I-FABP), total antioxidant capacity (T-AOC), and catalase (CAT) in piglet serum were measured using a commercial kit (Nanjing Jiancheng Bioengineering Institute, China). Inflammatory cytokines (IL-10, IL-1β, TNF-α, TGF-β) and apoptosis-related proteins (Caspase-3, Caspase-8, Caspase-9) in cell culture supernatants were quantified using ELISA kits (Meimian, China), following the manufacturer's instructions.

### Statistical analysis

Detailed methods for single-omic data analysis are described in the corresponding Method Details sections. For clinical and experimental data, standard statistical approaches were applied. Before conducting group comparisons, all continuous variables were tested for normality distribution using the Shapiro–Wilk test. Only data that met the normality assumption were subjected to further parametric analyses. Group comparisons were conducted using the Student’s *t*-test (for two groups) or one-way ANOVA followed by Tukey’s post hoc test (for multiple groups). For the formal cell culture experiments with a two-factorial design, statistical analysis was performed using two-way ANOVA to evaluate the main effects and their interaction. Post hoc comparisons were conducted using Tukey's multiple comparisons test. All statistical tests were two-tailed, and differences were considered statistically significant at *P* < 0.05.

For in vitro assays, each experiment was independently repeated at least three times. Results are presented as mean ± standard error of the mean (SEM). Graphs and statistical analyses were performed using GraphPad Prism version 5 (GraphPad Software, USA). Spearman’s rank correlation was used to evaluate associations between variables, with matched samples analyzed in a one-to-one manner.

## Result

### Oral administration of *P. copri* alleviates stress-induced diarrhea and colonic inflammation while enhancing intestinal integrity and antioxidant capacity in piglets

**Fig. 1:**
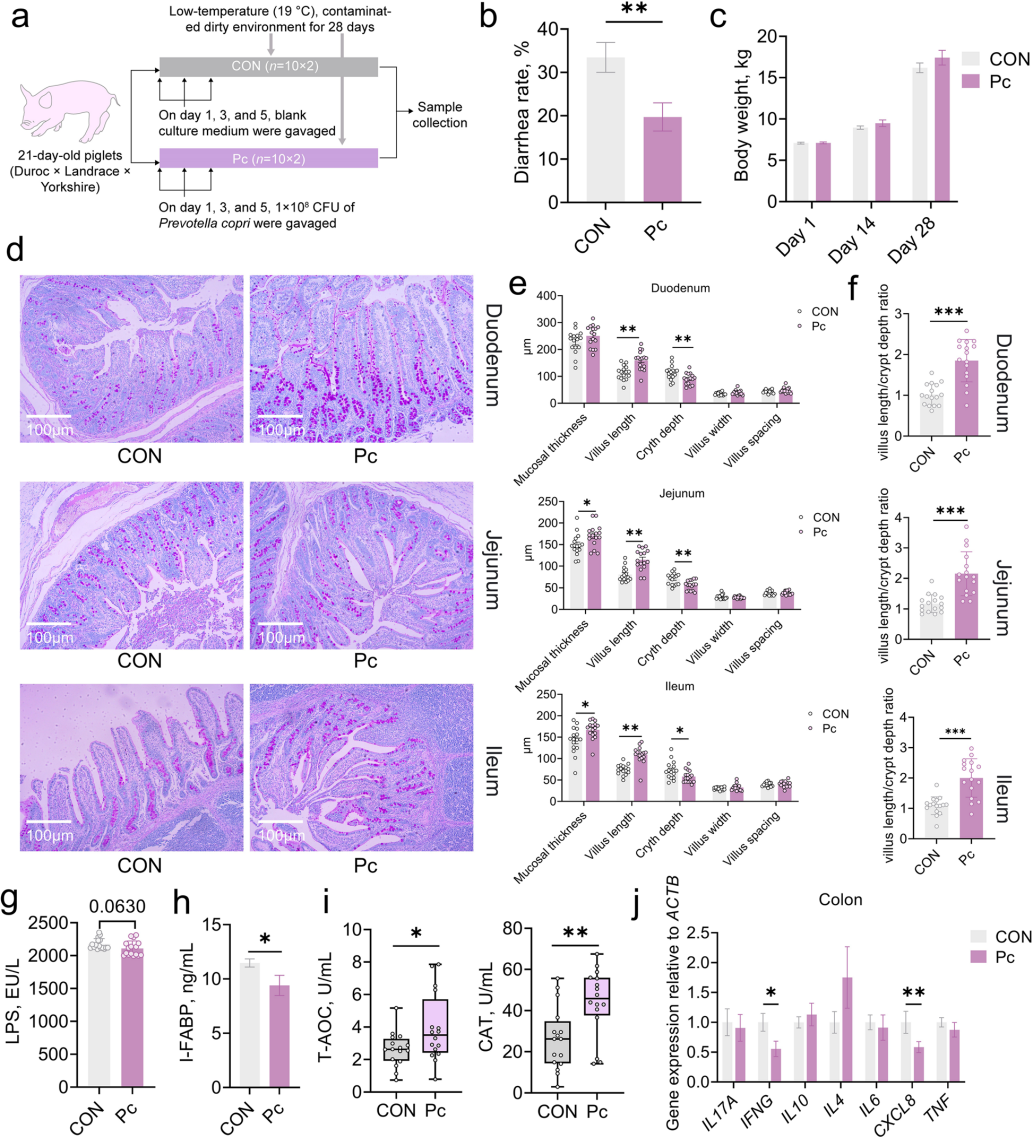
Effects of *P. copri* gavage on growth performance, diarrhea phenotype, intestinal morphology, serum biochemical parameters, and colonic inflammation in weaned piglets with stress-induced diarrhea. **a** Schematic diagram of animal trial. **b** Diarrhea rate of the weaned piglets throughout the experimental period.* n* = 10, each with 2 replicates. **c** Body weight of the weaned piglets at d 1, 14, and 28 of the experiment. *n* = 10, each with 2 replicates. **d** Morphology of the duodenal, jejunal and ileal mucosa in weaned piglets (PAS staining). **e** Morphometric parameters related to duodenal villus structure in the two groups.* n* = 8, each with 2 replicates. **f** Duodenal, jejunal and ileal villus length/crypt depth ratio in the two groups.* n* = 8, each with 2 replicates. **g** Level of lipopolysaccharides (LPS) in the serum of the weaned piglets. *n* = 8, each with 2 replicates. **h** Concentration of serum I-FABP in weaned piglets. *n* = 8, each with 2 replicates. **i** Serum T-AOC and CAT enzymatic activities in weaned piglets. *n* = 8, each with 2 replicates. **j** Relative expression of immune-related genes in the colon. *n* = 8, each with 2 replicates. All results are presented as the mean ± SEM. Statistical significance was assessed using a *t*-test, with significance levels indicated as follows: ^*^P < 0.05, ^**^P < 0.01, and ^***^P < 0.001

To induce stress-related diarrhea, piglets were exposed to a low-temperature and unhygienic pen environment (Fig. 1a). Under these conditions, the diarrhea rate was significantly higher in the control (CON) group compared to the *P. copri*-treated (Pc) group (Fig. 1b, *P* < 0.05), whereas no significant difference in body weight was observed between the two groups throughout the experimental period (Fig. 1c, *P* > 0.05). Histological analysis via H&E staining (Fig. 1d) revealed that *P. copri* administration significantly increased the mucosal thickness of the jejunum and ileum, elongated villi length, reduced crypt depth, and consequently elevated the villus-to-crypt ratio in the small intestine (Fig. 1e and f, *P* < 0.05). In addition, serum levels of lipopolysaccharide (LPS) and intestinal fatty acid-binding protein (I-FABP) were significantly decreased in the Pc group (Fig. 1g and h, *P* < 0.05), whereas the activities of total antioxidant capacity (T-AOC) and catalase (CAT) were markedly enhanced (Fig. 1i, *P* < 0.05). Compared to the CON group, the Pc group exhibited significantly lower MDA levels in the duodenal and jejunal mucosa (Table 1, *P* < 0.05), along with a marked increase in glutathione peroxidase GSH-Px activity in the ileal mucosa (Table 1, *P* < 0.05). Moreover, the mRNA expression levels of *IFNG* and *IL8* in the colonic mucosa were significantly downregulated in the Pc group compared with the CON group (Fig. 1j, *P* < 0.05).

**Table 1:** Antioxidant enzyme activity in intestinal mucosa^1^

Items	CON	Pc	*P*-value
Duodenum			
T-AOC, U/mgprot	1.08 ± 0.09	1.32 ± 0.20	0.309
T-SOD, U/mgprot	17.56 ± 1.18	19.27 ± 2.17	0.513
GSH-Px, U/mgprot	326.64 ± 20.49	382.63 ± 52.66	0.343
CAT, U/mgprot	13.00 ± 1.14	12.98 ± 1.10	0.986
MDA, nmol/mgprot	0.89 ± 0.09^a^	0.63 ± 0.05^b^	0.020
Jejunum			
T-AOC, U/mgprot	2.23 ± 0.23	2.01 ± 0.17	0.459
T-SOD, U/mgprot	30.06 ± 1.80	31.07 ± 2.48	0.756
GSH-Px, U/mgprot	386.36 ± 31.00	328.33 ± 19.69	0.126
CAT, U/mgprot	2.84 ± 0.45	4.83 ± 0.84	0.052
MDA, nmol/mgprot	1.00 ± 0.15^a^	0.50 ± 0.08^b^	0.010
Ileum			
T-AOC, U/mgprot	0.73 ± 0.17	0.93 ± 0.21	0.482
T-SOD, U/mgprot	22.01 ± 1.45	25.08 ± 1.01	0.093
GSH-Px, U/mgprot	600.95 ± 41.79^a^	768.90 ± 42.14^b^	0.009
CAT, U/mgprot	6.17 ± 0.99	6.51 ± 0.72	0.787
MDA, nmol/mgprot	0.57 ± 0.08	0.51 ± 0.08	0.515

^1^Data are expressed as mean ± SEM. Different superscript lowercase letters indicate statistically significant differences between groups (*P* < 0.05)

### Oral administration of *P. copri* increased fecal *Prevotella* abundance and reshaped the composition and functional profile of the colonic microbiota

**Fig. 2:**
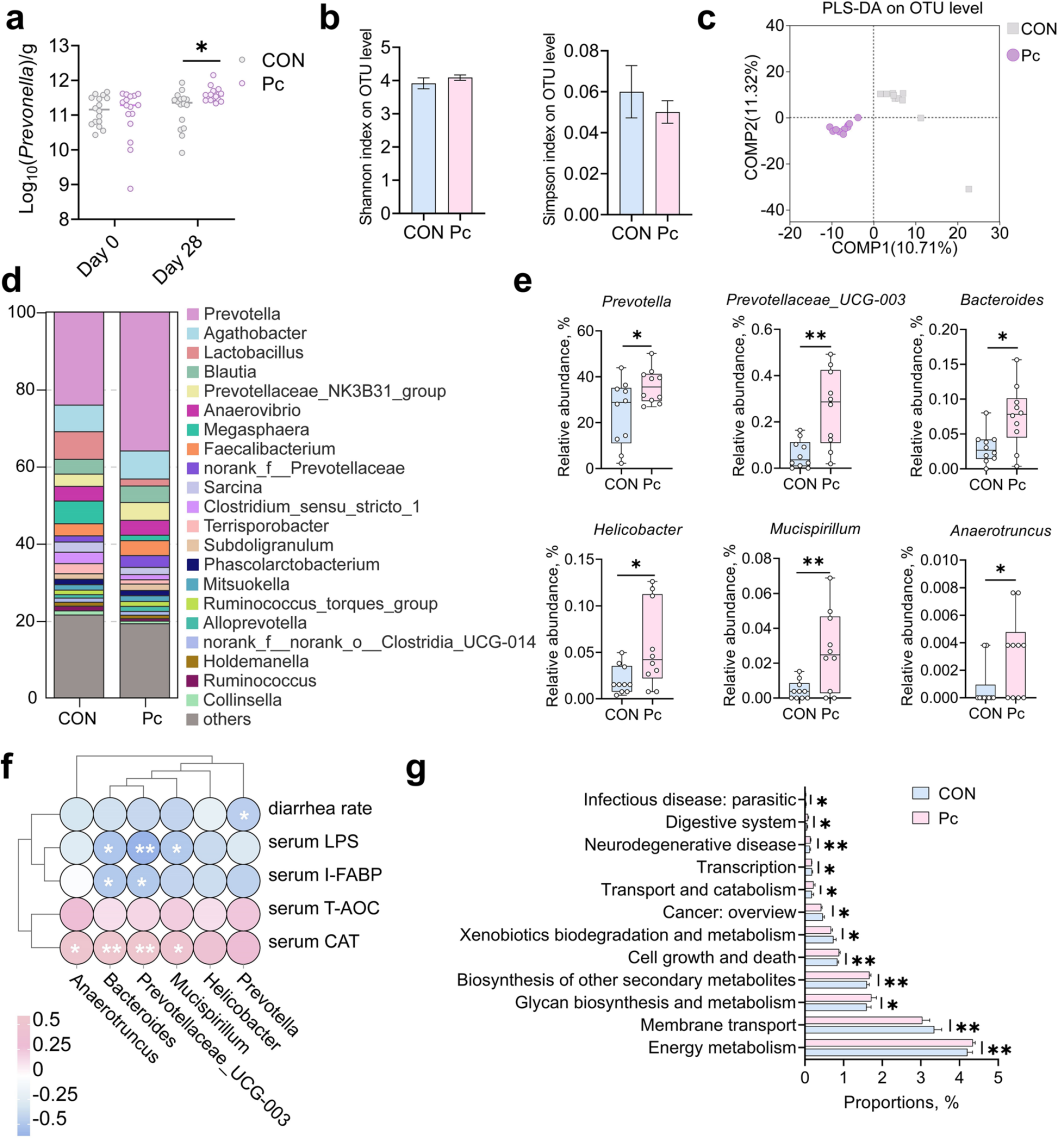
16S rRNA sequencing analysis of the colonic digesta microbiota in weaned piglets from the two groups. **a** Changes in the quantity of *Bacteroides* genus in the feces of the weaned piglets. *n* = 8, each with 2 replicates. **b** Microbial α-diversity indices based on 16S rRNA sequencing analysis. **c** artial least squares discriminant analysis (PLS-DA) of microbial communities at the OTU level based on 16S rRNA sequencing. **d** Stacked bar chart showing the relative abundances of the top 21 genera in the CON and Pc groups. **e** Box plots of the relative abundances of six significantly upregulated differential genera in the Pc group. **f** Heatmap of Spearman correlations between the six differential genera and piglet phenotypic parameters. **g** Bar plot of 12 significantly altered KEGG metabolic pathways between the two groups. ^*^P < 0.05, ^**^P < 0.01

Three oral doses of *P. copri* administered on d 1, 3, and 5 were sufficient to significantly increase the absolute abundance of *Prevotella* in feces by d 28 compared to the CON group (Fig. 2a, *P* < 0.05). 16S rRNA sequencing of colonic digesta revealed no significant differences in α-diversity, as measured by the Shannon and Simpson indices (Fig. 2b, *P* > 0.05). Nevertheless, partial least squares discriminant analysis (PLS-DA) showed a clear separation in microbial community composition between the two groups (Fig. 2c). The PCoA plot indicates a trend of separation between the control (CON) and treatment (Pc) groups (Fig. S1), however, the PERMANOVA test showed that this difference was not statistically significant (R^2^ = 0.076, *P* = 0.057, Table S4). Taxonomic profiling at the genus level revealed marked shifts in microbial composition following *P. copri* administration. A stacked bar chart depicting the top 21 genera in colonic digesta showed clear differences between the control (CON) and *P. copri*-treated (Pc) piglets (Fig. 2 d). Welch's *t*-test identified six genera that were significantly enriched in the Pc group, including *Prevotella*, *Prevotellaceae_UCG-003*, *Bacteroides*, *Helicobacter*, *Mucispirillum*, and *Anaerotruncus* (Fig. 2e, *P* < 0.05). Spearman correlation analysis between these genera and host phenotypes revealed that *Prevotella* was significantly negatively correlated with diarrhea rate. Both *Bacteroides* and *Prevotellaceae_UCG-003* were negatively associated with serum LPS and I-FABP levels, while positively correlated with serum CAT activity. *Mucispirillum* also showed a negative correlation with serum LPS and a positive correlation with serum CAT. In addition, *Anaerotruncus* was positively correlated with serum CAT levels (Fig. 2f, *P* < 0.05). Functional prediction based on PICRUSt2 indicated that *P. copri* administration significantly reshaped the metabolic functional landscape of the colonic microbiota. Compared to controls, piglets treated with *P. copri* exhibited higher predicted abundances of pathways associated with energy metabolism, membrane transport, glycan biosynthesis and metabolism, biosynthesis of secondary metabolites, and cell growth and death (Fig. 2g, Table S5, *P* < 0.05).

### Microbial ecological network analysis indicated enhanced stability and greater modularity in the co-occurrence network of the Pc group

**Fig. 3:**
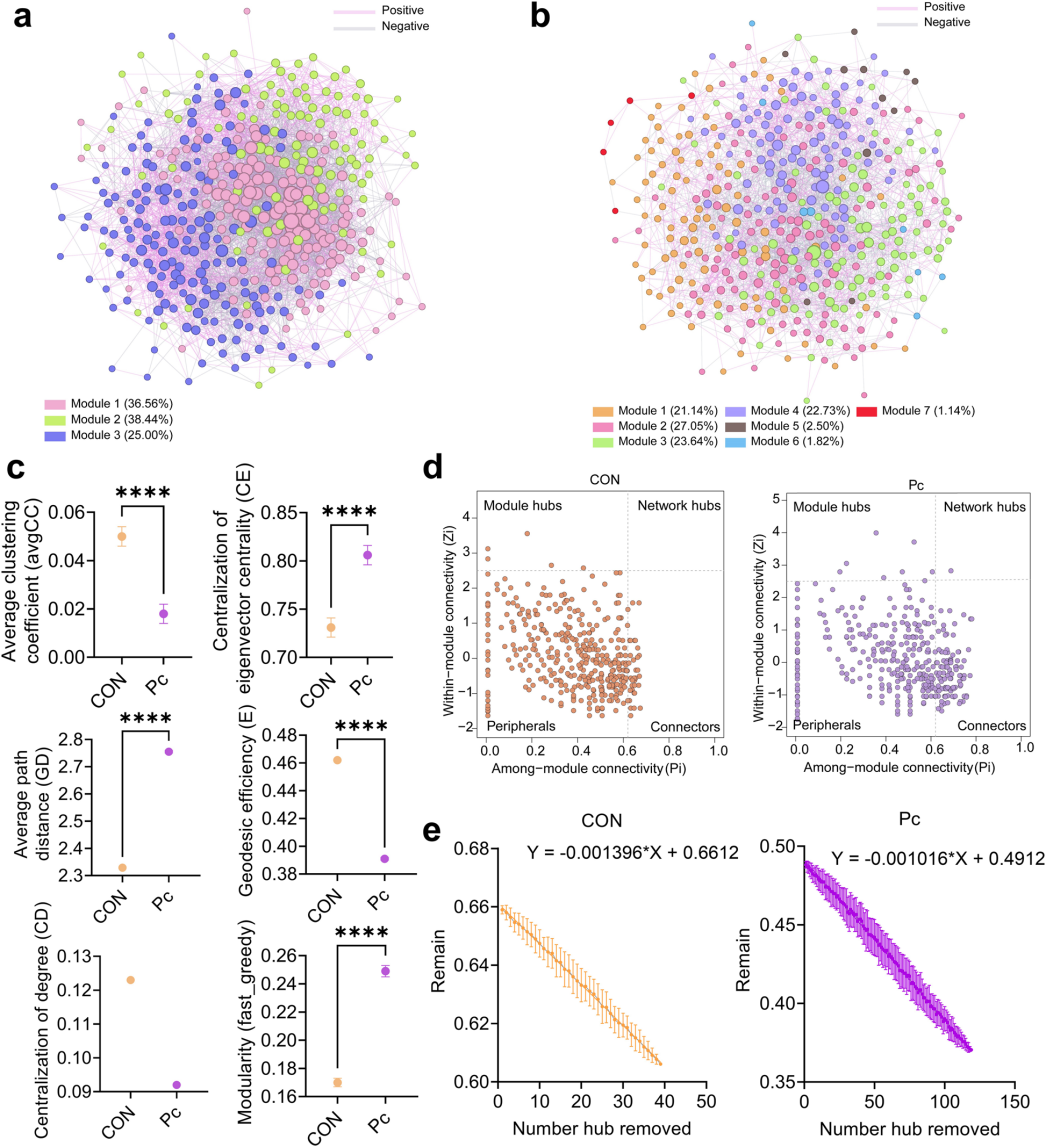
Microbial ecological networks in the colonic digesta of weaned piglets from the two groups. **a** and **b** Visualization of microbial ecological co-occurrence networks in colonic digesta from the CON and *P. copri* groups. Each node represents an operational taxonomic unit (OTU), and different colors indicate distinct modules. Networks were visualized using Gephi. Pink edges represent positive correlations, while gray edges represent negative correlations between OTUs. **c** Comparison of key topological properties of the networks between groups. ^****^P < 0.0001. **d** Node classification in the CON group network to identify potential keystone OTUs. Each symbol represents an OTU. Nodes with Zi > 2.5 and Pi > 0.62 are defined as network hubs; Zi > 2.5 and Pi ≤ 0.62 as module hubs; Zi ≤ 2.5 and Pi > 0.62 as connectors; and Zi ≤ 2.5 and Pi ≤ 0.62 as peripherals. **e** Robustness analysis of networks based on random removal of hub nodes. The proportion of remaining nodes reflects network robustness

Microbial ecological network analysis (MENs) revealed distinct topological structures between the CON and Pc groups (Fig. 3a and b). The CON network comprised 424 nodes and 4,440 edges, while the Pc network contained 440 nodes and 2,572 edges. The CON group was partitioned into three modules, whereas the Pc group exhibited higher modular complexity with six distinct modules. Analysis of the global network properties revealed substantial topological differences between the CON and Pc groups (Table S6). The co-occurrence network analysis revealed distinct differences in global network properties between the CON and Pc groups. The CON group exhibited a more complex and densely connected network structure, as reflected by a greater total number of links (4,440 vs. 2,572), higher average degree (20.94 vs. 11.69), increased density (0.0495 vs. 0.0266), and higher average clustering coefficient (0.285 vs. 0.247). Additionally, the CON network showed a shorter average path distance (2.60 vs. 3.05) and higher geodesic efficiency (0.422 vs. 0.359), indicating more efficient connectivity among nodes. In terms of centralization metrics, the CON network demonstrated higher centralization of degree (0.123 vs. 0.092), centralization of betweenness (0.026 vs. 0.042), and centralization of stress centrality (0.466 vs. 0.383), suggesting a stronger reliance on specific hub nodes, such as OTU163. Conversely, the Pc group exhibited a more decentralized and modular topology, with higher maximal betweenness (4,459.87 vs. 2,644.74) and greater centralization of eigenvector centrality (0.859 vs. 0.806), indicating a broader distribution of node influence and potential network resilience. Notably, although the Pc network was less densely connected, it exhibited slightly higher efficiency (0.976 vs. 0.953), suggesting that its more distributed architecture may support effective communication while minimizing vulnerability associated with central hubs.

Comparison with randomly generated networks (Table S6) showed that the Pc group had significantly lower average clustering coefficient (avgCC) and geodesic efficiency (E) than the CON group (Fig. 3c, *P* < 0.05), and a numerically lower centralization of degree (CD). In contrast, the Pc group displayed significantly higher centralization of eigenvector centrality (CE), average path distance (GD), and modularity (fast_greedy) than the CON group (Fig. 3c, *P* < 0.05). Following greedy modularity optimization, we assessed within-module connectivity (Z) and among-module connectivity (P) to identify network roles (Table S6, Fig. 3d). In the CON group, 5 module hubs, 33 connectors, and 386 peripheral species were detected. The Pc group, in comparison, showed 7 module hubs, 108 connectors, and 1 network hub. To assess network robustness, key hubs were sequentially removed based on Z-P values. Linear regression analysis revealed a steeper decline in the CON network (*Y* = −0.001396 × *X* + 0.6612) than in the Pc network (*Y* = −0.001016 × *X* + 0.4912), indicating a more gradual decrease in the relative size of the largest connected component in the Pc group (Fig. 3e).

### *P. copri*-derived metabolites drive antibiotic biosynthesis and antimicrobial activity while oral gavage enriches arachidonic acid metabolism in piglet colonic digesta

Given the significant increase in both the absolute and relative abundance of *Prevotella* following oral administration of *P. copri*, along with its marked impact on colonic microbial composition and ecological network structure, we further explored the metabolic features of cultured *P. copri* and its effects on colonic chyme metabolites in piglets. Untargeted metabolomics was conducted on cell-free supernatants from *P. copri* monoculture and the corresponding blank culture medium. Principal component analysis (PCA) revealed a clear separation between *P. copri* and control samples along both PC1 and PC2 axes, indicating substantial differences in their metabolic profiles (Fig. S2a).

Volcano plot visualization of differentially abundant metabolites showed that, in the negative ion mode, 1,527 metabolites were upregulated and 1,651 were downregulated in the *P. copri* group compared with controls. In the positive ion mode, 2,109 metabolites were upregulated and 1,427 were downregulated (Fig. S2b). KEGG pathway enrichment analysis was performed on the differentially abundant metabolites, and the top 15 enriched pathways were visualized (Fig. S2c). The most significantly enriched categories were "Metabolic pathways" (784 metabolites), "Biosynthesis of secondary metabolites" (725 metabolites), and "Biosynthesis of antibiotics" (602 metabolites).

Based on these findings, we further examined the antimicrobial effects of *P. copri* metabolites against three common porcine intestinal pathogens. Co-incubation with *P. copri* supernatant significantly reduced the pathogens’ maximum growth rate and prolonged the lag phase, compared to the control group (Fig. S2d–f, *P* < 0.05). Furthermore, PI fluorescence analysis, using CTAB as a positive control, revealed increased membrane permeability in ETEC upon co-incubation with *P. copri* metabolites, with a significant enhancement observed after 30 min (Fig. S2g, *P* < 0.05).

**Fig. 4:**
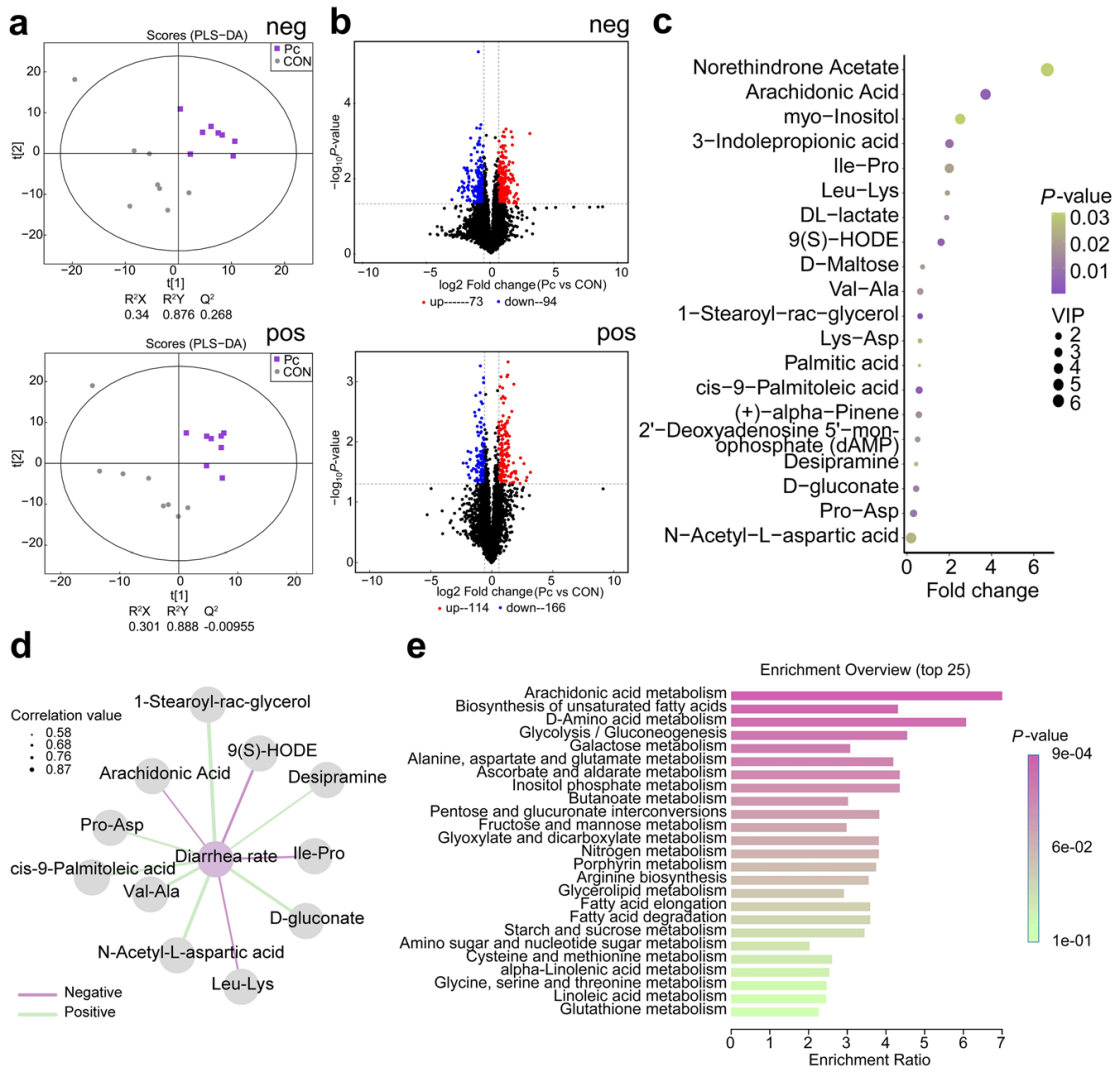
Non-targeted metabolomic profiling of colonic digesta in weaned piglets from the two groups. **a** Partial least squares discriminant analysis (PLS-DA) of metabolites in colonic digesta. CON: piglets gavaged with blank culture medium; Pc: piglets gavaged with *P. copri* culture medium. Pos: positive ion mode; Neg: negative ion mode. R^2^X represents the explained variance in predictor variables; R^2^Y represents the explained variance in response variables; Q^2^Y indicates model predictability. **b** Volcano plots of metabolite differences between groups. Metabolites with fold change > 1.5 and *P* < 0.05 are shown in magenta; those with fold change < 0.67 and *P* < 0.05 are shown in blue; non-significant metabolites are shown in black. **c** Significantly altered metabolites were identified by combining multivariate analysis (Variable Importance in Projection, VIP > 1) with univariate statistical testing (*P* < 0.05). **d** Correlation network between differential metabolites and phenotypic traits based on Spearman correlation (*P* < 0.05 and |correlation coefficient| > 0.5). Positive correlations are shown as green solid lines, and negative correlations as purple dashed lines. **e** Top 25 enriched KEGG pathways in group Pc compared to the CON group, based on differential metabolites. The node size indicates the enrichment ratio, calculated as Hits/Expected (where Hits = number of observed hits, Expected = number of expected hits)

In the piglet experiment, oral administration of *P. copri* led to a trend toward increased levels of AA and BA in colonic digesta (Table 2, *P* < 0.01). To further investigate metabolic changes, untargeted metabolomic profiling was conducted to compare the metabolic composition between the CON and *P. copri* groups. Partial least squares discriminant analysis (PLS-DA) revealed clear separation between the Pc group and the CON group in both negative (neg) and positive (pos) ion modes, indicating distinct metabolic profiles between the groups. The high R^2^Y values (> 0.87) in both models suggest strong explanatory power for the grouping variables (Fig. 4a). Compared to the CON group, 73 metabolites were upregulated and 94 were downregulated in the negative ion mode, while 114 metabolites were upregulated and 166 downregulated in the positive ion mode (Fig. 4b). Based on orthogonal PLS-DA (OPLS-DA), differential metabolites were identified using variable importance in projection (VIP) scores. The top 20 metabolites were visualized in a bubble plot, with the highest-ranked being norethindrone acetate, arachidonic acid, and myo-inositol (Fig. 4c). Spearman correlation analysis was performed to associate differential metabolites with diarrhea incidence in piglets, revealing six metabolites significantly positively correlated and three negatively correlated with diarrhea rates, including arachidonic acid, 9(S)-HODE, and Leu-Lys (Fig. 4d). KEGG enrichment pathway analysis of differential metabolites identified arachidonic acid metabolism as the most significantly enriched metabolic pathway, with the highest enrichment ratio and lowest *P* value (Fig. 4e).

**Table 2:** Short-chain fatty acid (SCFA) content in colonic digesta^1^

Items	CON	Pc	*P*-value
AA, mmol/g	6.16 ± 0.91	7.93 ± 0.34	0.093
PA, mmol/g	4.00 ± 0.18	3.94 ± 0.29	0.869
IBA, mmol/g	0.16 ± 0.03	0.17 ± 0.02	0.814
BA, mmol/g	0.43 ± 0.05	0.58 ± 0.05	0.062
IVA, mmol/g	0.14 ± 0.02	0.15 ± 0.02	0.768
VA, mmol/g	0.39 ± 0.11	0.31 ± 0.09	0.569
Total SCFA, mmol/g	11.28 ± 1.02	13.07 ± 0.49	0.136

^1^Data are expressed as mean ± SEM. Different superscript lowercase letters indicate statistically significant differences between groups (*P* < 0.05)

### Extracted metabolites of *P. copri* alleviate TNF-α-induced inflammatory damage and enhance gene expression related to AHR–Nrf2 pathway in Caco-2 cells

**Fig. 5:**
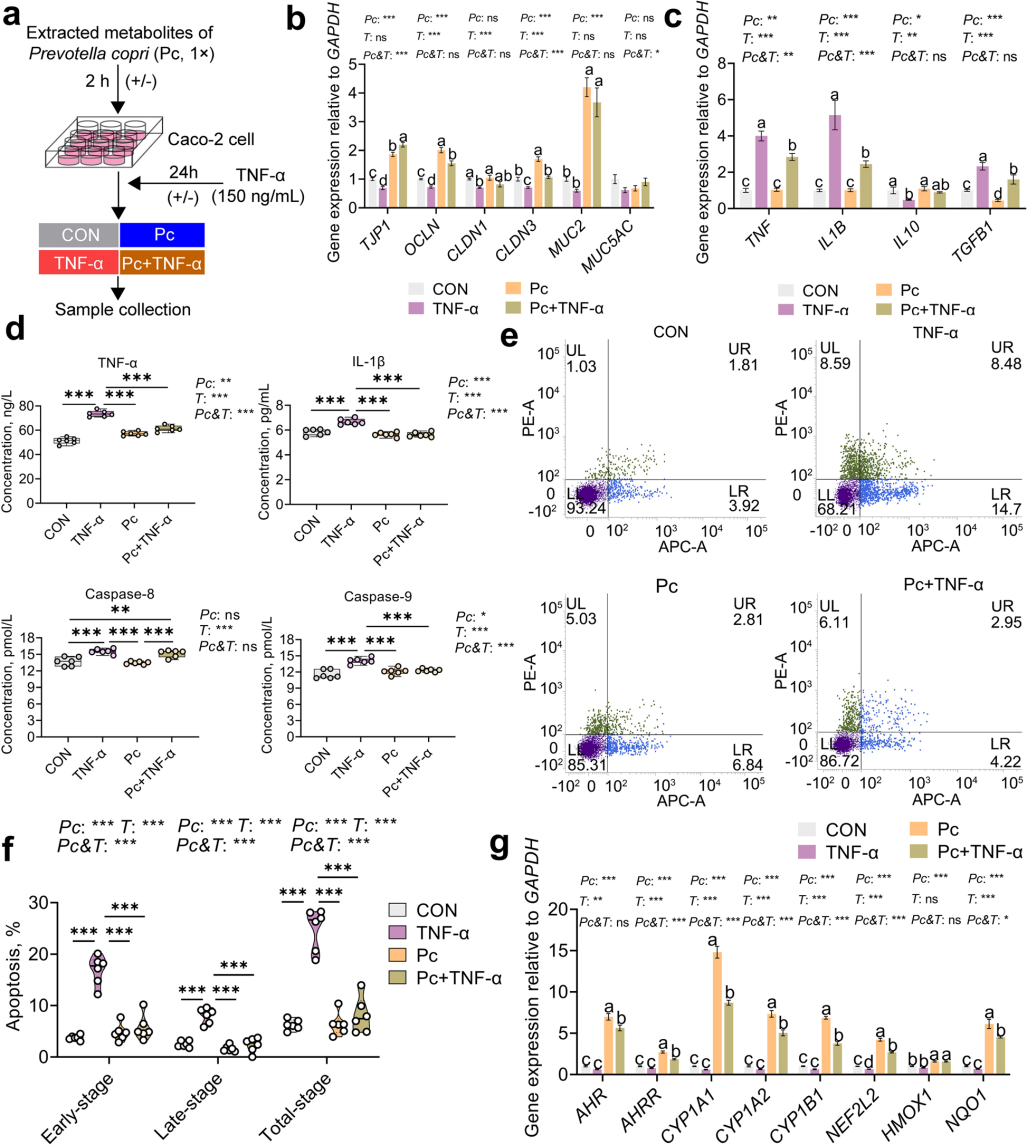
Effect of *P. copri* metabolites on TNF-α-induced intestinal barrier function, inflammation, and apoptosis in Caco-2 cells. **a** Schematic diagram of experimental design. **b** Relative expression of intestinal barrier-related genes in each group. **c** Relative expression of inflammation-related genes in each group. **d** Concentration of inflammatory cytokines and apoptosis-related proteins detected by ELISA. **e** Representative flow cytometry profiles of different groups. Each plot is divided into four quadrants: UL, necrotic cells; UR, cells in the late stage of apoptosis; LR, cells in the early stage of apoptosis; LL, normal cells. **f** The early-stage, late-stage and total apoptosis rate (early + late) of Caco-2 cells in different groups. **g** Relative expression levels of AHR–Nrf2 signaling pathway-related genes in each group. CON: Caco-2 cells treated with PBS for 26 h,* n* = 6; TNF-α: Caco-2 cells treated with PBS for 2 h, then treated with 150 ng/mL TNF-α for 24 h,* n* = 6; Pc: Caco-2 cells treated with 1 × extracted metabolites of *P. copri* for 2 h, then treated with PBS for 24 h,* n* = 6; Pc + TNF-α: Caco-2 cells treated with 1 × extracted metabolites of *P. copri* for 2 h, then treated with 150 ng/mL TNF-α for 24 h,* n* = 6. All data are presented as the mean ± SEM. Statistical significance was assessed by two-way ANOVA for the main effects of *Pc* (administration of *P. copri* metabolites), *T* (TNF-α challenge), and their interaction (*Pc&T*). Tukey's honestly significant difference (HSD) test was used to determine significant differences among multiple groups. ^a–d^Different letters indicate significant differences (*P* < 0.05). ^*^P < 0.05, ^**^P < 0.01, ^***^P < 0.001. All results were obtained from three independent experiments

Metabolomic profiling highlighted a strong association between arachidonic acid metabolism by the colonic microbiota and the growth performance and diarrhea phenotype in weaned piglets. To further investigate the underlying mechanisms, an inflammatory model was established using TNF-α-challenged Caco-2 cells (Fig. 5a). The concentration of *P. copri*-derived metabolites was set at 1 ×, which corresponds to the equivalent concentration present in the original bacterial culture supernatant. At this concentration, the metabolites did not affect the viability of Caco-2 cells (Fig. S3a, *P* > 0.05), but significantly enhanced the viability of TNF-α-challenged cells (Fig. S3b, *P* < 0.05), upregulated the expression of genes associated with intestinal barrier function, and downregulated the expression of pro-inflammatory cytokines and apoptosis-related genes (Fig. S3c, *P* < 0.05). These findings were used to assess whether *P. copri*-derived metabolites modulate the inflammatory response via tryptophan-associated signaling pathways.

A two-factor experimental model was established using Caco-2 cells pretreated with *P. copri*-derived metabolites followed by TNF-α stimulation (Fig. 5a). Two-way ANOVA revealed significant main effects of *P. copri*-derived metabolites (Pc) and TNF-α (T), as well as their interaction (Pc&T), across multiple parameters. For intestinal barrier-related genes, *P. copri*-derived metabolites significantly affected the expression of *TJP1*, *OCLN*, *CLDN3*, and *MUC2* (*P* < 0.001; Fig. 5b), while TNF-α exerted a significant main effect on *OCLN*, *CLDN1*, and *CLDN3* (*P* < 0.001; Fig. 5b). Significant interaction effects were observed for *TJP1*, *CLDN3*, and *MUC5AC* (*P* < 0.05; Fig. 5b). Post hoc comparisons further showed that TNF-α markedly reduced the expression of *TJP1*, *OCLN*, *CLDN1*, and *CLDN3* compared to the control group (*P* < 0.05; Fig. 5b), whereas *P. copri*-derived metabolites significantly upregulated *TJP1*, *OCLN*, *CLDN3*, and *MUC2* (*P* < 0.05). Co-treatment with *P. copri* and TNF-α significantly restored the expression of *TJP1*, *OCLN*, *CLDN3*, and *MUC2* compared to TNF-α alone (*P* < 0.05; Fig. 5b).

For pro-inflammatory cytokines (*TNF* and *IL1B*), both main effects and interaction effects were highly significant (*P* < 0.01; Fig. 5c). Multiple comparisons indicated that *P. copri*-derived metabolites significantly suppressed TNF-α-induced upregulation of these genes (*P* < 0.05; Fig. 5c). In contrast, anti-inflammatory genes (*IL10* and *TGFB1*) were significantly influenced by the main effects of both *P. copri*-derived metabolites and TNF-α (*P* < 0.05), while no significant interaction was observed (*P* > 0.05; Fig. 5c). Post hoc analysis showed that TNF-α significantly downregulated *IL10* and upregulated *TGFB1* expression compared to the control (*P* < 0.05; Fig. 5c).

ELISA assays confirmed these trends at the protein level, revealing significant main and interaction effects on the secretion of TNF-α, IL-1β, and Caspase-9 (*P* < 0.05; Fig. 5d). Multiple comparisons indicated that TNF-α significantly increased the secretion of these proteins (*P* < 0.05; Fig. 5d), while both *P. copri* alone and in combination with TNF-α significantly reduced their levels compared to TNF-α alone (*P* < 0.05; Fig. 5d). Flow cytometry analysis of apoptosis (Fig. 5e–f) showed significant main and interaction effects (*P* < 0.001; Fig. 5f), with *P. copri*-derived metabolites significantly attenuating TNF-α-induced apoptosis.

Finally, for genes involved in the AHR–Nrf2 signaling pathway, *P. copri*-derived metabolites had a strong main effect on all examined genes (*AHR*, *AHRR*, *CYP1A1*, *CYP1A2*, *CYP1B1*, *NFE2L2*, *HMOX1*, and *NQO1*) (*P* < 0.001; Fig. 5g). TNF-α also had a significant main effect on all genes except *HMOX1* (*P* < 0.001; Fig. 5g). Notably, significant interaction effects were observed for all genes except *AHR* and *HMOX1* (*P* < 0.001; Fig. 5g). Multiple comparisons showed that both the *P. copri* and *P. copri* + TNF-α groups had significantly higher expression of AHR–Nrf2-related genes compared to the control and TNF-α groups (*P* < 0.05; Fig. 5g).

### Arachidonic acid alleviates TNF-α-induced inflammatory damage in colonic epithelial cells via the AHR–Nrf2 signaling pathways

**Fig. 6:**
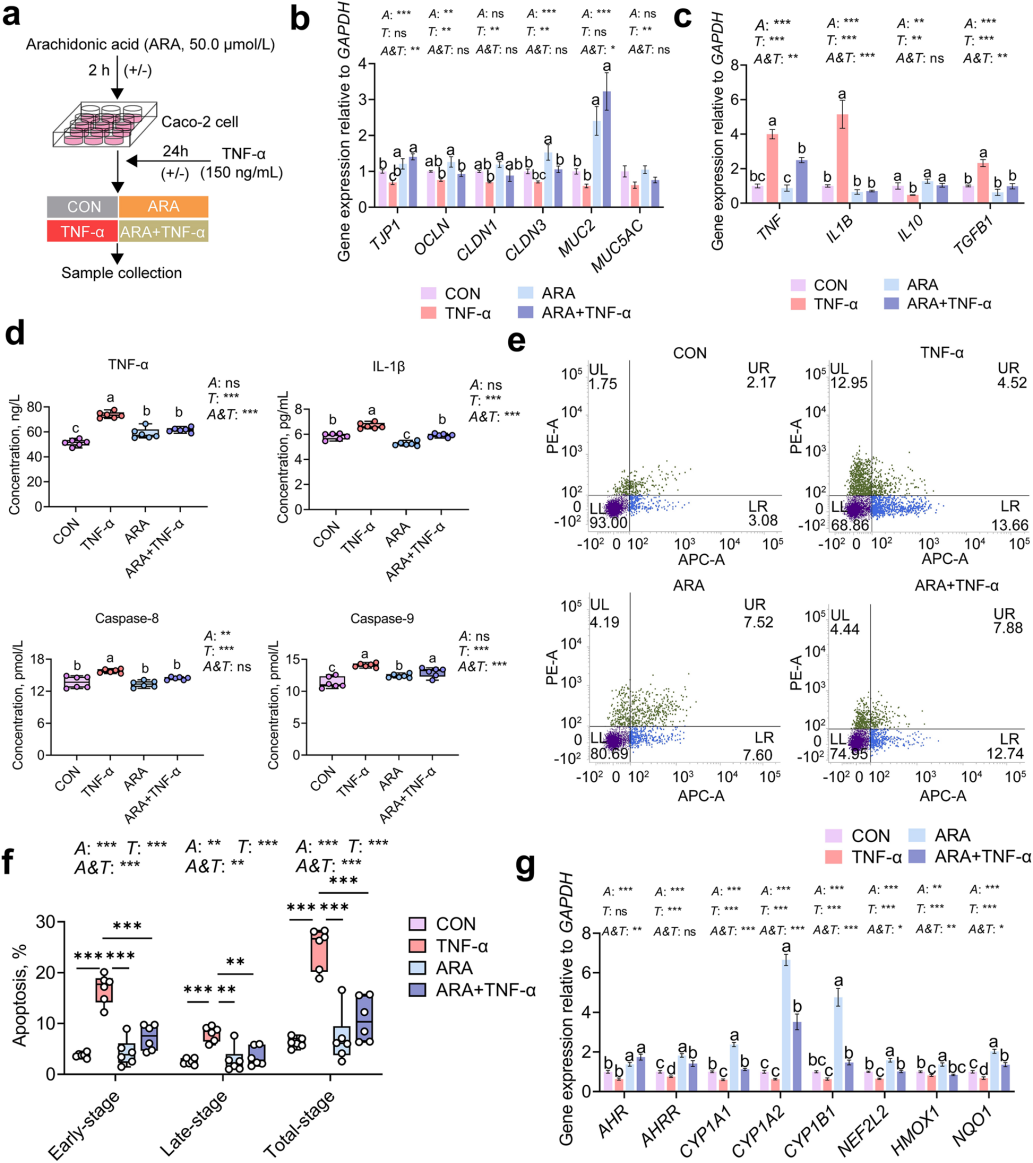
Effect of arachidonic acid on TNF-α-induced intestinal barrier function, inflammation, and apoptosis in Caco-2 cells. **a** Schematic diagram of experimental design. **b** Relative expression of intestinal barrier-related genes in each group. **c** Relative expression of inflammation-related genes in each group. **d** Concentration of inflammatory cytokines and apoptosis-related proteins detected by ELISA. **e** Representative flow cytometry profiles of different groups. Each plot is divided into four quadrants: UL, necrotic cells; UR, cells in the late stage of apoptosis; LR, cells in the early stage of apoptosis; LL, normal cells. **f** The early-stage, late-stage and total apoptosis rate (early + late) of Caco-2 cells in different groups. **g** Relative expression levels of AHR–Nrf2 signaling pathway-related genes in each group. CON: Caco-2 cells treated with PBS for 26 h,* n* = 6; TNF-α: Caco-2 cells treated with PBS for 2 h, then treated with 150 ng/mL TNF-α for 24 h,* n* = 6; AA: Caco-2 cells treated with 50 µmol/L arachidonic acid for 2 h, then treated with PBS for 24 h,* n* = 6; AA + TNF-α: Caco-2 cells treated with 50 µmol/L arachidonic acid for 2 h, then treated with 150 ng/mL TNF-α for 24 h,* n* = 6. All data are presented as the mean ± SEM. Statistical significance was assessed by two-way ANOVA for the main effects of *A* (administration of ARA), *T* (TNF-α challenge), and their interaction (*A&T*). Tukey's honestly significant difference (HSD) test was used to determine significant differences among multiple groups. ^a–d^Different letters indicate significant differences (*P* < 0.05). ^*^P < 0.05, ^**^P < 0.01, *P* < 0.001. All results were obtained from three independent experiments

Based on untargeted metabolomic profiling, the ARA metabolism pathway was found to be significantly enriched in the Pc group, and the metabolites of *P. copri* were observed to alleviate TNF-α-induced inflammation in Caco-2 cells. To further test the hypothesis that ARA mediates the observed anti-inflammatory effects, we established a co-incubation model in which Caco-2 cells were pretreated with ARA (50 μmol/L) prior to TNF-α stimulation (Fig. 6b). At this concentration, ARA did not affect the viability of untreated Caco-2 cells (Fig. S4a, *P* > 0.05), but significantly improved the viability of TNF-α-challenged cells (Fig. S4b, *P* < 0.05). In addition, ARA pretreatment upregulated the expression of genes related to intestinal barrier integrity and downregulated the expression of pro-inflammatory cytokines and apoptosis-related genes (Fig. S4c, *P* < 0.05).

A two-factor experimental model was established using Caco-2 cells pretreated with ARA followed by TNF-α stimulation (Fig. 6a). Two-way ANOVA revealed significant main effects of ARA on the expression of key intestinal barrier-related genes, including *TJP1*, *OCLN*, *CLDN3*, and *MUC2* (*P* < 0.01, Fig. 6b). TNF-α also exerted strong main effects on most intestinal barrier genes (*P* < 0.01, Fig. 6b), except for *OCLN* and *MUC5AC*. Notably, significant interaction effects between ARA and TNF-α were observed for *TJP1* and *MUC2* (*P* < 0.01, Fig. 6b), and post hoc analysis confirmed that ARA significantly upregulated their expression under TNF-α stimulation compared to the CON and TNF-α groups (*P* < 0.05).

Significant main effects of ARA (A) and TNF-α (T) were also observed for inflammatory cytokines *TNF*, *IL1B*, *IL10*, and *TGFB1* (*P* < 0.01, Fig. 6c), with notable interaction effects for *TNF*, *IL1B*, and *TGFB1* (*P* < 0.01). Multiple comparisons showed that ARA significantly downregulated the expression of *TNF*, *IL1B*, and *TGFB1* under TNF-α stimulation (*P* < 0.01, Fig. 6c).

ELISA results for inflammation- and apoptosis-related proteins revealed significant main effects of TNF-α on TNF-α, IL-1β, Caspase-8, and Caspase-9 levels (*P* < 0.01, Fig. 6 d). Significant ARA × TNF-α interaction effects were found for TNF-α, IL-1β, and Caspase-9 (*P* < 0.01, Fig. 6 d), with the ARA + TNF-α group showing significantly lower concentrations compared to the TNF-α group (*P* < 0.01, Fig. 6d).

Flow cytometry analysis further showed that ARA pre-treatment significantly alleviated TNF-α-induced apoptosis (Fig. 6e). Two-way ANOVA revealed significant main and interaction effects on early, late, and total apoptosis rates (*P* < 0.001, Fig. 6f). Multiple comparisons demonstrated that TNF-α significantly increased apoptosis across all stages compared to other groups (*P* < 0.001, Fig. 6f).

Finally, analysis of AHR–Nrf2 signaling-related gene expression revealed strong main effects of ARA on all examined genes (*AHR*, *AHRR*, *CYP1A1*, *CYP1A2*, *CYP1B1*, *NFE2L2*, *HMOX1*, and *NQO1*) (*P* < 0.001, Fig. 6g). TNF-α also showed significant main effects on all genes except *AHR* (*P* < 0.001, Fig. 6g). Significant interaction effects were observed for all genes except *AHRR* (*P* < 0.05, Fig. 6g). Post hoc comparisons indicated that AHR–Nrf2 pathway genes were significantly upregulated in the ARA group compared to all other groups (*P* < 0.05, Fig. 6g), and that co-treatment with ARA and TNF-α significantly increased the expression of *AHR*, *CYP1A1*, *CYP1A2*, *CYP1B1*, *NFE2L2*, and *NQO1* relative to TNF-α alone (*P* < 0.05, Fig. 6g), suggesting enhanced activation of the AHR–Nrf2 axis under inflammatory stress.

## Discussion

Despite extensive research, diarrhea in humans and neonatal piglets remains a clinical challenge [26], further complicated by rising antibiotic resistance due to widespread antibiotic use [27]. Our previous meta-analysis identified *Prevotella* as the third most abundant genus in piglets by two weeks of age, which significantly increases post-weaning and eventually dominates the gut microbiota [8]. Notably, its relative abundance is higher in healthy piglets compared to those with diarrhea, suggesting a potential role in maintaining gut health [28]. Based on this, we used a weaning-stress piglet model to explore the association between *P. copri* colonization, diarrhea rate, and microbial homeostasis.

To establish a stress-induced model mimicking post-weaning diarrhea, piglets were exposed to low ambient temperature and unhygienic housing conditions, resulting in diarrhea rates comparable to those observed on commercial farms without antimicrobial intervention [29]. Oral administration of *P. copri* for three consecutive doses significantly enriched *Prevotella* in the gut by d 28, thereby establishing a *Prevotella*-dominant microbiota in weaned piglets. *P. copri* intervention markedly reduced diarrhea incidence and lowered serum levels of LPS and I-FABP under stress conditions. These improvements were likely attributed to enhanced intestinal morphology and barrier integrity, reduced intestinal inflammation, and improved systemic antioxidant capacity, as evidenced by increased T-AOC and CAT activities.

Oral administration of *P. copri* modulated the colonic microbiota of weaned piglets under cold stress. Although *P. copri* gavage did not significantly affect α-diversity and only showed a slight impact on β-diversity, we further explored its effects on the gut microbiota. Differential abundance analysis revealed an increase in *Prevotella*. To understand its potential role, we performed correlation analysis with host serum phenotypes, KEGG functional prediction, and microbial network analysis. *Prevotella* remained the dominant genus in both groups, but its absolute and relative abundances were significantly increased in *P. copri*-treated piglets, indicating successful colonization and sustained activity in the gut. Notably, only *Prevotella*, *Prevotellaceae_UCG-003*, *Bacteroides*, *Helicobacter*, *Mucispirillum*, and *Anaerotruncus* were closely associated with the improved phenotypes observed. Among these, *Prevotella*, *Prevotellaceae_UCG-003*, and *Bacteroides* are known for their capacity to degrade dietary polysaccharides and fibers [30–32]; *Mucispirillum* specializes in utilizing host-derived mucins [33]; and *Anaerotruncus* is a butyrate-producing anaerobe [34]. These genera are important contributors to the production of microbial secondary metabolites, which may play key roles in host gut health and stress adaptation.

In addition to differences in microbial composition and specific taxa, we further investigated the impact of *P. copri* gavage on the ecological co-occurrence network of the gut microbiota. The gut microbial community forms a complex ecological network that is critical for host health, and the introduction of probiotics can induce significant ecological and evolutionary shifts within this system [35, 36]. In diarrheal piglets under stress, the gut microbial co-occurrence network exhibited a more compact and centralized topology, characterized by a greater reliance on hub nodes and thus increased fragility. In contrast, *P. copri* administration resulted in a more decentralized and modular architecture with slightly reduced propagation efficiency but enhanced ecological robustness. These findings were supported by random network modeling. Furthermore, simulations of random hub removal revealed that the gut ecological network of diarrheal piglets disintegrated more rapidly, highlighting its increased susceptibility to node loss due to high centralization and reliance on keystone taxa such as OTU163. In contrast, the gut network of piglets administered *P. copri* exhibited a slower decline in connectivity, reflecting greater structural redundancy and enhanced resilience to perturbations. These topological features suggest that microbial interactions in the *P. copri*-treated group are more robust against ecological disruptions, with no single taxon disproportionately dominating network cohesion. Modularity, often driven by natural selection, is critical for maintaining community stability [37], and the increased number of modules following probiotic intervention may contribute to the resilience of the gut ecosystem in this context [38, 39]. Notably, the stability of microbial networks upon hub node loss is considered a key factor in sustaining microbial homeostasis [40]. These results suggest that *P. copri* may influence gut function and host health by modulating specific microbes and their interactions, even without major changes in overall diversity.

The gut commensal microbiota influences host physiology through nutrient- and metabolite-dependent mechanisms, wherein microbial metabolites directly interact with intestinal epithelial and immune cells to modulate host health [41]. In the mucosal niche, commensals also compete with pathogens by producing antimicrobial compounds [42]. Unexpectedly, metabolic profiling of *P. copri* culture supernatants revealed significant enrichment of pathways related to antibiotic biosynthesis. Subsequent validation confirmed the presence of antimicrobial metabolites capable of inhibiting pathogenic growth and viability. This finding suggests that future in vivo studies could investigate the protective and therapeutic effects of *P. copri* against pathogenic infections in the small intestine. Metabolomic analysis of colonic digesta from *P. copri*-treated piglets further identified ARA as a differentially abundant metabolite, which was negatively correlated with diarrhea incidence and significantly enriched in the ARA metabolism pathway. Consistently, previous studies have demonstrated that *P. copri* treatment increased fecal ARA levels in mice [43], and that high abundance of *P. copri* in the pig intestine was associated with elevated serum ARA levels [16]. These similar observations further support the notion that oral administration of *P. copri* in the present study is closely linked to the enrichment of the arachidonic acid metabolic pathway in the feces. Previous studies have shown that ARA supplementation alleviated ER stress and protected the colonic mucosa in *Il10*^*−/−*^ mice [44]. Additionally, ARA has been reported to suppress NLRP3 inflammasome activation by inhibiting phospholipase C and reducing JNK signaling [45]. Collectively, these findings suggest that ARA may serve as a microbial-derived mediator facilitating host–microbiota communication in the gut.

In this study, metabolites derived from *P. copri* attenuated TNF-α-induced inflammation and apoptosis by activating the AHR–Nrf2 signaling axis, while also promoting the expression of genes encoding tight junction proteins and mucins. Notably, significant interaction effects were observed between *P. copri*-derived metabolites and TNF-α, indicating that these metabolites exert enhanced anti-inflammatory effects under inflammatory conditions. AHR activation is known to induce the transcription of phase I and phase II xenobiotic-metabolizing enzymes, including *CYP1A1*, *CYP1A2*, and *CYP1B1* [46]. Nrf2 has been identified as a key downstream effector in the AHR signaling cascade [47]. Notably, *P. copri* metabolites upregulated the mRNA levels of both *AHR* and *AHRR*, and enhanced the enzymatic activity of associated pathways. This activation subsequently triggered Nrf2 induction, leading to the increased expression of antioxidant genes such as *HMOX1* and *NQO1* [48–50]. In addition, these metabolites modulated LPS-induced inflammation and conferred protection against oxidative stress in both cells and tissues [51]. Collectively, these findings demonstrate that *P. copri* metabolites exert protective effects on colonic epithelial cells by mitigating oxidative stress and inflammation through activation of the AHR–Nrf2 pathway. Emerging evidence suggests that endogenous ARA and its derivatives can serve as ligands for the aryl hydrocarbon receptor (AHR), thereby initiating AHR–chaperone complex signaling cascades [52, 53]. In particular, lipoxin A4 (LXA4), a LOX-derived metabolite of ARA, has been identified as a potent AHR activator with anti-inflammatory potential [54]. While direct investigations into the effects of ARA on intestinal inflammation remain limited, our study demonstrates that exogenous ARA supplementation alleviates inflammatory responses in colonic epithelial cells, at least in part via activation of the AHR–Nrf2 signaling pathway, with a pronounced interaction observed between ARA and TNF-α. Although ARA has traditionally been viewed as a pro-inflammatory fatty acid due to its role in producing eicosanoids [55], recent evidence suggests a more nuanced function in the intestinal environment. ARA and its metabolites (e.g., PGE₂, Lipoxins, 15-HETE) have been shown to exert anti-inflammatory effects in the gut, including suppression of pro-inflammatory cytokines, enhancement of epithelial repair, and modulation of immune responses [56]. Several in vivo studies further support its protective role: dietary ARA reduced ER stress, oxidative damage, and fibrosis in *Il10*^*−/−*^ mice [44], and did not exacerbate colitis in DSS models, except at very high doses [57]. Additionally, ARA and its metabolites can promote intestinal epithelial cell proliferation via receptor-mediated pathways [58]. These findings indicate that ARA, at appropriate physiological levels, may help maintain intestinal homeostasis, aligning with our observations of its anti-inflammatory effects both in vitro and in vivo. These findings provide new insights into the interplay between ARA and AHR in intestinal homeostasis, although the underlying mechanisms warrant further elucidation. It should also be noted that only a single strain of *P. copri* was evaluated in this study. Further research employing isolation and culture techniques is needed to investigate the specific functions and efficacy of pig-derived *P. copri* strains in weaned piglets. 

## Conclusion

In summary, our study demonstrates that oral administration of *Prevotella copri* markedly improves intestinal health and reduces diarrhea incidence in weaned piglets under stress, and further explores the underlying mechanisms. *P. copri* supplementation enhances intestinal morphology and barrier integrity while alleviating inflammation. Mechanistically, *P. copri* modulates gut microbial composition and ecological network interactions, leading to a more dispersed yet modular and robust microbial architecture. Additionally, *P. copri* alters the colonic metabolite profile, among which arachidonic acid was identified as a key metabolite that enhances epithelial barrier function, reduces apoptosis, and mitigates inflammatory immune responses via activation of the AHR–Nrf2 signaling pathway. These findings not only establish *P. copri* as a critical contributor to maintaining gut health in weaned piglets, but also highlight its potential as a candidate therapeutic agent for intestinal disorders such as neonatal diarrhea. 

## Supplementary Information

Additional file 1: Table S1 The ingredients and nutritional levels of feed (as-fed basis). Table S2 Standard of diarrhea scoring. Table S3 The sequences of primers used for the qPCR analysis in the animal experiment.

Additional file 2: Table S4 PERMANOVA results based on Bray-Curtis distances showing differences in microbial community structure between the CON and Pc groups.

Additional file 3: Table S5 Differential analysis of predicted KEGG pathway abundances between CON and Pc groups based on 16S rRNA gene sequencing data.

Additional file 4: Table S6 Properties of molecular ecological networks (MENs) in CON and Pc groups based on RMT.

Additional file 5: Fig. S1 Principal Coordinates Analysis (PCoA) of gut microbiota based on OTU-level Bray–Curtis distances. Fig. S2 Untargeted metabolomic profiling of *P. copri* monoculture and the inhibitory effects of its extracted metabolites on pathogenic bacteria. Fig. S3 Determination of the co-culture concentration of P. copri metabolite extracts. Fig. S4 Effects of different dosage of arachidonic acid treatment on the mRNA level of tight junction protein, inflammatory factor, cell apoptosis-related gene of cells treated with TNF-α.

## Data Availability

The sequencing data and mass spectrometry data reported in this paper have been deposited in the Genome Warehouse in National Genomics Data Center (NGDC), Beijing Institute of Genomics, Chinese Academy of Sciences/China National Center for Bioinformation under the BioProject accession number PRJCA023182 that is publicly accessible at https://www.ngdc.cncb.ac.cn/bioproject[59, 60]. All the clean genome sequencing data were deposited in the Genome Sequence Archive (GSA) of NGDC under the accession number CRA023403. The metabolome data of the single-strain culture and fecal metabolome data have been deposited in the Open Archive for Miscellaneous Data (OMIX) at the National Genomics Data Center (NGDC) under accession numbers OMIX008866 and OMIX008869.

## Abbreviations

ARA: Arachidonic acid
AHR: Aryl hydrocarbon receptor
ANOVA: Analysis of variance
ATCC: American type culture collection
CAT: Catalase
Caspase-3: Cysteine-aspartic acid protease 3
Caspase-8: Cysteine-aspartic acid protease 8
Caspase-9: Cysteine-aspartic acid protease 9
Caco-2: Human colorectal adenocarcinoma cell line
CTAB: Cetyltrimethylammonium bromide
ELISA: Enzyme-linked immunosorbent assay
ETEC: Enterotoxigenic *E**scherichia* *coli*
HMDB: Human metabolome database
i-FABP: Intestinal fatty acid binding protein
IL-1β: Interleukin-1 beta
IL-10: Interleukin-10
KEGG: Kyoto Encyclopedia of Genes and Genomes
LB: Luria–Bertani medium
LPS: Lipopolysaccharide
MS/MS: Tandem mass spectrometry
mzXML: Mass spectrometry data format (XML-based)
NRC: National Research Council
Nrf2: Nuclear factor erythroid 2-related factor 2
OTU: Operational taxonomic unit
PCA: Principal component analysis
PBS: Phosphate-buffered saline
PCR: Polymerase chain reaction
*P. copri*: *Prevotella copri*
PI: Propidium iodide
PLS-DA: Partial least squares discriminant analysis
RMT: Random matrix theory
SEM: Standard error of the mean
T-AOC: Total antioxidant capacity
TGF-β: Transforming growth factor beta
TNF-α: Tumor necrosis factor alpha
UPLC-Q-TOF/MS: Ultra-performance liquid chromatography coupled with quadrupole time-of-flight mass spectrometry
VIP: Variable importance in projection
