# Supplementary material for: Prevotella copri alleviates diarrhea in weaning piglets through gut microbiota modulation and arachidonic acid–AHR–NRF2 pathway activation
Source: J Anim Sci Biotechnol. 2025 Nov 20;16:154. doi: 10.1186/s40104-025-01273-y (PMC12632048; doi:10.1186/s40104-025-01273-y)
Supplement: Supplementary file 1 — Additional file 1: Table S1 The ingredients and nutritional levels of feed (as-fed basis). Table S2 Standard of diarrhea scoring. Table S3 The sequences of primers used for the qPCR analysis in the animal experiment. [file 40104_2025_1273_MOESM1_ESM.docx]

**Table S1** The ingredients and nutritional levels of feed (as-fed basis)

| **Ingredient** | **g/kg** |
| --- | --- |
| Corn | 396.00 |
| Corn (extruded) | 130.00 |
| SBM (high protein, 47%) | 70.00 |
| Soy protein concentrate | 70.00 |
| Soybean (extruded) | 90.00 |
| Whey | 100.00 |
| Fish meal | 60.00 |
| Glucose | 50.00 |
| Limestone | 8.50 |
| MCP | 5.00 |
| NaCl | 2.50 |
| Soy bean oil | 6.00 |
| Premix^1^ | 5.00 |
| L-Lys·HCl | 4.70 |
| DL-Met | 0.80 |
| Thr | 1.30 |
| Trp | 0.20 |
| Total, kg | 1000.00 |
| Nutritional level^2^ |  |
| ME, kcal/kg | 3396 |
| CP, % | 20.36 |
| Total Ca, % | 0.79 |
| Total P, % | 0.64 |
| SID, % |  |
| Lys | 1.33 |
| Met | 0.39 |
| Met+Cys | 0.63 |
| Thr | 0.75 |
| Trp | 0.21 |
| Val | 0.79 |

^1^ Premixes provide the following per kg of feed: vitamin A, 9,750 IU; vitamin D_3_, 3,000 IU; vitamin E, 63 mg; vitamin K_3_, 3.0 mg; vitamin B_1_, 3.0 mg; vitamin B_2_, 9.6 mg; vitamin B_6_, 4.5 mg; vitamin B_12_, 36 μg; D-biotin, 240 μg; D-calcium pantothenate, 30 mg; folic acid, 1.8 mg; niacin, 36 mg; Cu (tribasic copper chloride), 190 mg; I (potassium iodate), 0.6 mg; Fe (ferrous sulfate), 120 mg; Mn (manganese sulfate), 60 mg; Zn (zinc sulfate), 120 mg; Se (sodium selenite), 450 μg; choline (choline chloride), 300 mg; and Ca (calcium carbonate) 0.6 g

^2^ Nutrient levels are calculated values

**Table S2** Standard of diarrhea scoring

| **Fecal scores** | **Feces characteristic** | **Evaluation** |
| --- | --- | --- |
| 0 | Hard or pellet-like feces | Normal |
| 1 | Normal feces, soft and well-formed | Mild diarrhea |
| 2 | Loose feces, partially formed | Moderate diarrhea |
| 3 | Semi-liquid feces, watery stool | Severe diarrhea |

**Table S3** The sequences of primers used for the qPCR analysis in the animal experiment

| **Targeting gene** | **Primer sequence （5’ to 3’）** | **References/Accession No.** |
| --- | --- | --- |
| *Sus scrofa* |  |  |
| *ACTB* | F: TCTGGCACCACACCTTC | NM_001101 |
|  | R: TGATCTGGGTCATCTTC |  |
| *GAPDH* | F: TTTGCGTCAGTGTCATCG  R: TGCTCTGCCTTGGGTAAT | [1] |
| *IFNG* | F: TCTAACCTAAGAAAGCGGAAGAGA | NM_213948.1 |
|  | R: TTGCAGGCATGACAATTA |  |
| *IL10* | F: CCTGGAAGACGTAATGCCGA | NM_214041.1 |
|  | R: CACGGCCTTGCTCTTGTTTT |  |
| *IL17A* | F: CCCTGTCACTGCTGCTTCTG | NM_001005729.1 |
|  | R: TCATGATTCCCGCCTTCAC |  |
| *IL4* | F: GGACACAAGTGCGACATCA | [2] |
|  | R: GCACGTGTGGTGTCTGTA |  |
| *IL6* | F: AGGGAAATGTCGAGGCTGTGC | [3] |
|  | R: CCGGCATTTGTGGTGGGGTT |  |
| *CXCL8* | F: TAGGACCAGAGCCAGGAAGA | [4] |
|  | R: AATTTGGGGTGGAAAGGTGT |  |
| *TNF* | F: CGTGAAGCTGAAAGACAACCAG | [5] |
|  | R: GATGGTGTGAGTGAGGAAAACG |  |
| *Homo sapiens* |  |  |
| *GADPH* | F: GTCGGAGTGAACGGATTTGG | [6] |
|  | R: CAATGTCCACTTTGCCAGAGTTAA |  |
| *TJP1* | F: ATCCCTCAAGGAGCCATTC | [7] |
|  | R: CACTTGTTTTGCCAGGTTTTA |  |
| *MUC2* | F: CAGCACCGATTGCTGACTTG | [7] |
|  | R: GCTGGTCATCTCAATGGCAG |  |
| *OCLN* | F: CCAATGTCGAGGAGTGGG | [7] |
|  | R: CGCTGCTGTAACGAGGCT |  |
| *CLDN1* | F: GCATGAAGTGTATGAAGTGCTTGGA | [8] |
|  | R: CGATTCTATTGCCATACCATGCTG |  |
| *CLDN3* | F: CACGCGAGAAGAAGTACACG | [9] |
|  | R: GTAGTCCTTGCGGTCGTAGC |  |
| *MUC5AC* | F: CGACCTGTGCTGTGTACCAT | [6] |
|  | R: CCACCTCGGTGTAGCTGAA |  |
| *TNF* | F: CTGCCTGCTGCACTTTGGAG | [10] |
|  | R: ACATGGGCTACAGGCTTGTCACT |  |
| *IL1B* | F: CCAGGGACAGGATATGGAGCA | [10] |
|  | R: TTCAACACGCAGGACAGGTACAG |  |
| *TGFB1* | F: AGGGCTACCATGCCAACTTC | [11] |
|  | R: GCGGCACGCAGCACTGAT |  |
| *IL10* | F: GTGATGCCCCAAGCTGAGA | [12] |
|  | R: CACGGCCTTGCTCTTGTTTT |  |
| *AHR* | F: TGGGTCCAGTCTAATGCACG | [13] |
|  | R: TGCTCTGTTCCTTCCTCATCT |  |
| *CYP1A1* | F: GTGATCCCAGGCTCCAAGAG | [13] |
|  | R: AGAAGAAACTCCGTGGCCG |  |
| *CYP1A2* | F: GCTGAATGGCTTCTACATCCCC | [13] |
|  | R: GCGGTGAGGAACCGCTC |  |
| *CYP1B1* | F: GCAGCGGAGATGAAAATGAGG | [13] |
|  | R: TTCCGATTCGCACAGACTGG |  |
| *AHRR* | F: GCAGCGGAGATGAAAATGAGG | [13] |
|  | R: TTCCGATTCGCACAGACTGG |  |
| *NFE2L2* | F: ATTGCTACTAATCAGGCTCAG | [13] |
|  | R: GTTTGGCTTCTGGACTTGG |  |
| *HMOX1* | F: ATGAACTCCCTGGAGATGACTC | [13] |
|  | R: CCTTGGTGTCATGGGTCAG |  |
| *NQO1* | F: CAATTCAGAGTGGCATTC | [13] |
|  | R: GAAGTTTAGGTCAAAGAGG |  |

**References**

1. Li G, Su H, Zhou Z, Yao W. Identification of the porcine G protein-coupled receptor 41 and 43 genes and their expression pattern in different tissues and development stages. PLoS One. 2014;9(5):e97342. https://doi.org/10.1371/journal.pone.0097342.

2. Nuntaprasert A, Mori Y, Muneta Y, Yoshihara K, Tsukiyama-Kohara K, Kai C. The effect of recombinant swine interleukin-4 on swine immune cells and on pro-inflammatory cytokine productions in pigs. Comp Immunol Microbiol Infect Dis. 2005;28:83–101. https://doi.org/10.1016/j.cimid.2004.07.002.

3. Xie K, Su G, Chen D, Yu B, Huang Z, Yu J, et al. The immunomodulatory function of the porcine β-defensin 129: Alleviate inflammatory response induced by LPS in IPEC-J2 cells. Int J Biol Macromol. 2021;188:473–81. https://doi.org/10.1016/j.ijbiomac.2021.07.194.

4. Yang T, Zhang F, Zhai L, He W, Tan Z, Sun Y, et al. Transcriptome of porcine PBMCs over two generations reveals key genes and pathways associated with variable antibody responses post PRRSV vaccination. Sci Rep. 2018;8:2460. https://doi.org/10.1038/s41598-018-20701-w.

5. Zhou H, Sun J, Ge L, Liu Z, Chen H, Yu B, et al. Exogenous infusion of short-chain fatty acids can improve intestinal functions independently of the gut microbiota. J Anim Sci. 2020;98(12):skaa371. https://doi.org/10.1093/jas/skaa371.

6. Nielsen DSG, Jensen BB, Theil PK, Nielsen TS, Knudsen KEB, Purup S. Effect of butyrate and fermentation products on epithelial integrity in a mucus-secreting human colon cell line. J Funct Foods. 2018;40:9–17. https://doi.org/10.1016/j.jff.2017.10.023.

7. Vernay T, Cannie I, Gaboriau F, Gall SD-L, Tamanai-Shacoori Z, Burel A, et al. *Bacteroides fragilis* prevents *Salmonella* Heidelberg translocation in co-culture model mimicking intestinal epithelium. Benef Microbes. 2020;11:391–401. https://doi.org/10.3920/bm2020.0004.

8. Wang W, Xia T, Yu X. Wogonin suppresses inflammatory response and maintains intestinal barrier function via TLR4-MyD88-TAK1-mediated NF-κB pathway in vitro. Inflamm Res. 2015;64:423–31. https://doi.org/10.1007/s00011-015-0822-0.

9. Feng W, Wu Y, Chen G, Fu S, Li B, Huang B, et al. Sodium butyrate attenuates diarrhea in weaned piglets and promotes tight junction protein expression in colon in a GPR109A-dependent manner. Cell Physiol Biochem. 2018;47:1617–29. https://doi.org/10.1159/000490981.

10. Hasegawa T, Mizugaki A, Inoue Y, Kato H, Murakami H. Cystine reduces tight junction permeability and intestinal inflammation induced by oxidative stress in Caco-2 cells. Amino Acids. 2021;53:1021–32. doi:10.1007/s00726-021-03001-y.

11. Liu S, Wang Y, Wen H, Sun X, Wang Y. Hydroxysafflor yellow A inhibits TNF-α-induced inflammation of human fetal lung fibroblasts via NF-κB signaling pathway. Evid Based Complement Alternat Med. 2019;2019:4050327. https://doi.org/10.1155/2019/4050327.

12. Białas M, Fiszer D, Rozwadowska N, Kosicki W, Jedrzejczak P, Kurpisz M. The role of IL-6, IL-10, TNF-alpha and its receptors TNFR1 and TNFR2 in the local regulatory system of normal and impaired human spermatogenesis. Am J Reprod Immunol. 2009;62:51–9. https://doi.org/10.1111/j.1600-0897.2009.00711.x.

13. Rueda GH, Causada-Calo N, Borojevic R, Nardelli A, Constante M, Loonen L, et al. A8 Dietary tryptophan modulates kynurenine and indole production in healthy individuals. J Can Assoc Gastroenterol. 2021;4(Suppl 1):9–10. https://doi.org/10.1093/jcag/gwab002.007.
