# Supplementary material for: Prevotella copri alleviates diarrhea in weaning piglets through gut microbiota modulation and arachidonic acid–AHR–NRF2 pathway activation
Source: J Anim Sci Biotechnol. 2025 Nov 20;16:154. doi: 10.1186/s40104-025-01273-y (PMC12632048; doi:10.1186/s40104-025-01273-y)
Supplement: Supplementary file 5 — Additional file 5: Fig. S1 Principal Coordinates Analysis (PCoA) of gut microbiota based on OTU-level Bray–Curtis distances. Fig. S2 Untargeted metabolomic profiling of P. copri monoculture and the inhibitory effects of its extracted metabolites on pathogenic bacteria. Fig. S3 Determination of the co-culture concentration of P. copri metabolite extracts. Fig. S4 Effects of different dosage of arachidonic acid treatment on the mRNA level of tight junction protein, inflammatory factor, cell apoptosis-related gene of cells treated with TNF-α. [file 40104_2025_1273_MOESM5_ESM.docx]

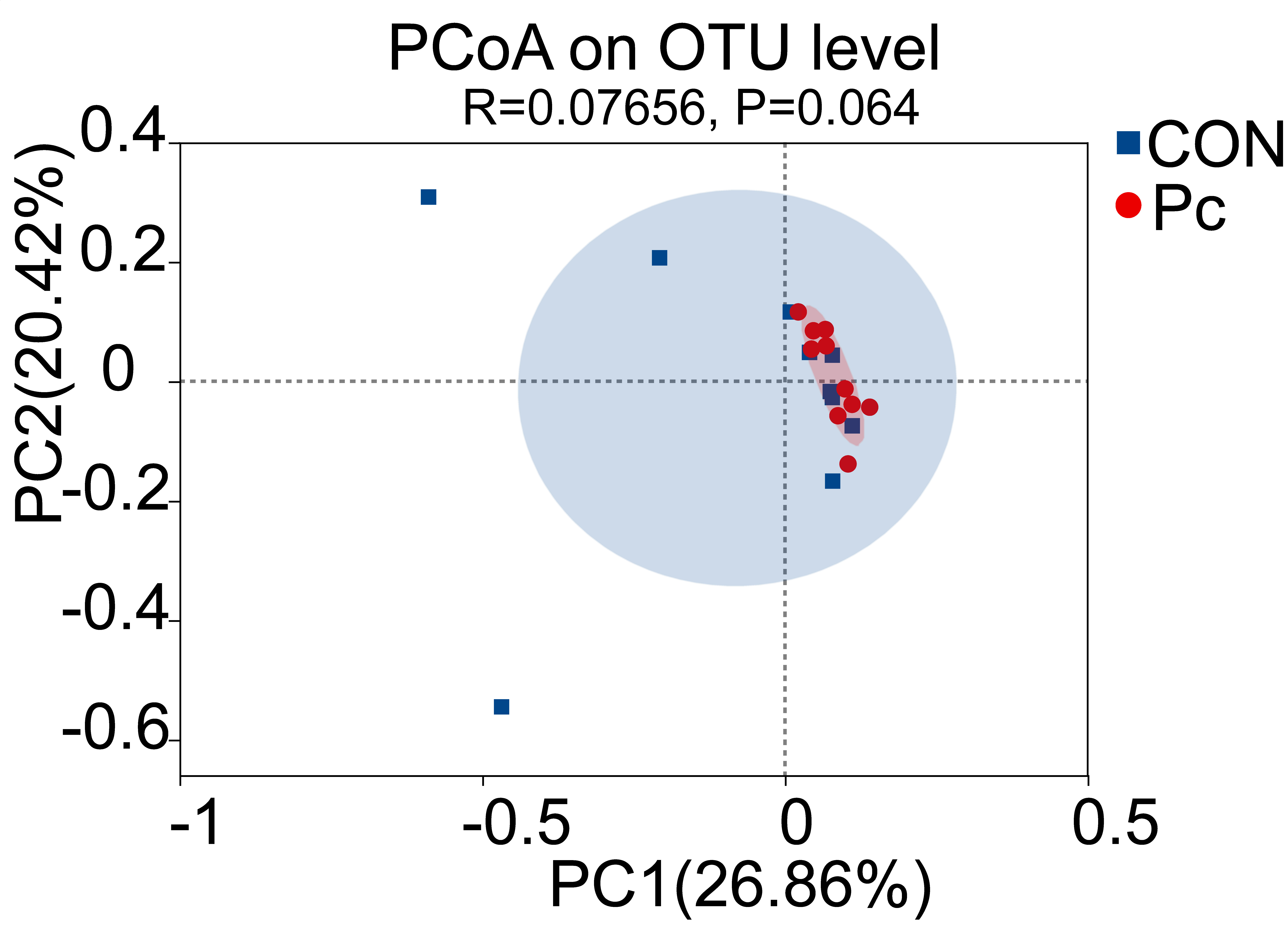
**Fig. S1** Principal Coordinates Analysis (PCoA) of gut microbiota based on OTU-level Bray–Curtis distances. PCoA was performed to assess β-diversity between the control (CON, blue squares) and *P. copri*-treated (Pc, red circles) groups. Each point represents the microbial community of an individual sample


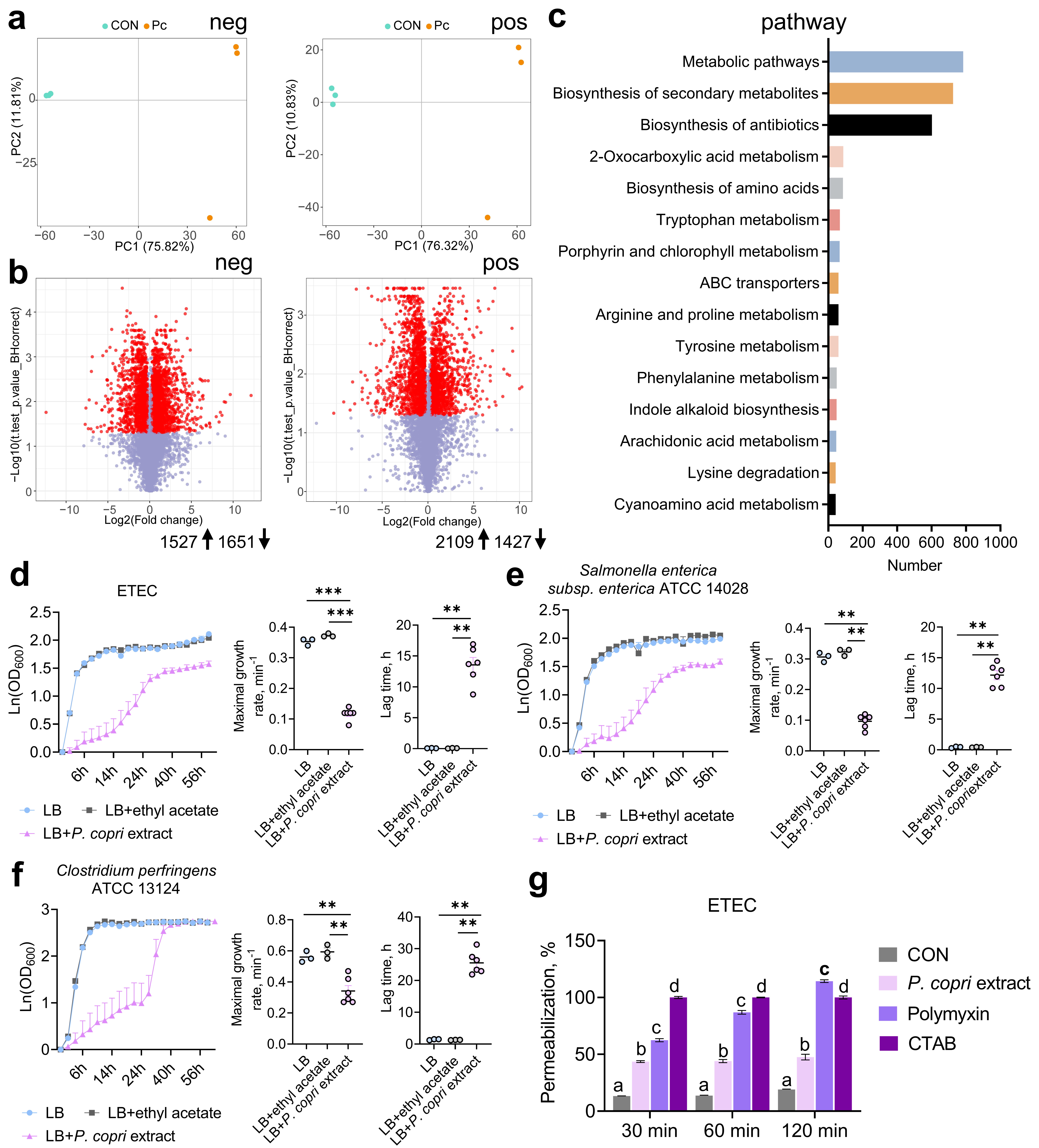
**Fig. S2** Untargeted metabolomic profiling of *P. copri* monoculture and the inhibitory effects of its extracted metabolites on pathogenic bacteria. **a** Principal component analysis (PCA) of metabolites in the culture medium. CON: blank medium without *P. copri* inoculation; Pc: *P. copri*-inoculated culture medium. Pos: positive ion mode; Neg: negative ion mode. **b** Volcano plot comparing metabolite profiles between the Pc and CON groups. Metabolites with a fold change ≤ 0.8333 or ≥ 1.2 and *P* < 0.05 are highlighted in red, while non-significant metabolites are shown in blue. **c** KEGG pathway classification of the top 15 significantly altered metabolites in the *P. copri* culture medium. “Number” refers to the number of metabolites annotated to each corresponding pathway. **d–f** Effects of *P. copri*-derived metabolites on the growth of Enterotoxigenic *Escherichia coli* (ETEC), *Salmonella enterica* subsp. *enterica* ATCC 14028, and *Clostridium perfringens* ATCC 13124. Growth curves were recorded for each treatment group: LB (negative control, basal medium + pathogen, *n* = 3), LB + ethyl acetate extract (positive control, basal medium + pathogen + ethyl acetate extract, *n* = 3), and LB + *P. copri* extract (basal medium + pathogen + *P. copri*-derived metabolites, *n* = 6). Scatter plots display (1) the maximum growth rate and (2) the time to reach the logarithmic growth phase in each group. **g** Membrane permeabilization assay of ETEC co-incubated with *P. copri*-derived metabolites or reference compounds. Groups include: CON (ETEC cultured in basal medium, *n* = 6), *P. copri* extract (ETEC cultured with *P. copri*-derived metabolites, *n* = 6), Polymyxin (ETEC cultured with polymyxin, *n* = 6), and CTAB (ETEC cultured with cetyltrimethylammonium bromide, *n* = 6). All data are presented as mean ± SEM. Statistical differences among groups were analyzed using Tukey’s Honestly Significant Difference (HSD) test. ^**^*P* < 0.01; different superscript letters indicate statistically significant differences at *P* < 0.05


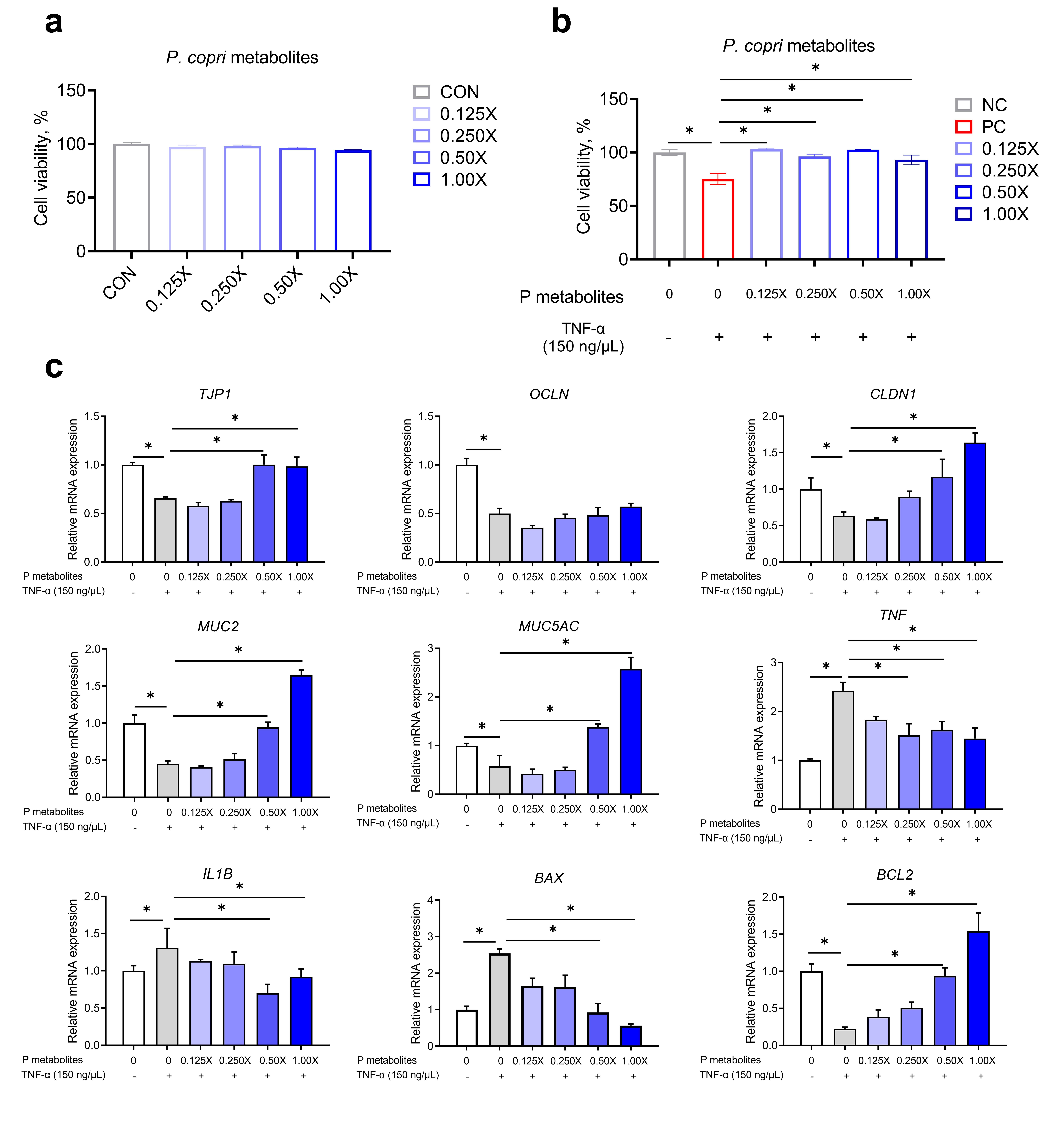


**Fig. S3** Determination of the co-culture concentration of *P. copri* metabolite extracts. **a** The effect of different concentrations of *P. copri* metabolite extracts on the viability of Caco-2 cells after 24 h. **b** The impact of different concentrations of *P. copri* metabolite extracts on TNF-α-induced viability of Caco-2 cells. NC: negative control, PC: positive control. **c** The effect of different concentrations of *P. copri* metabolite extracts on the relative expression levels of genes related to intestinal barrier, inflammation, and apoptosis in TNF-α-induced Caco-2 cells. ^*^*P* < 0.05. The biological replicates is *n* = 4, obtained from two independent experiments using different cell passages


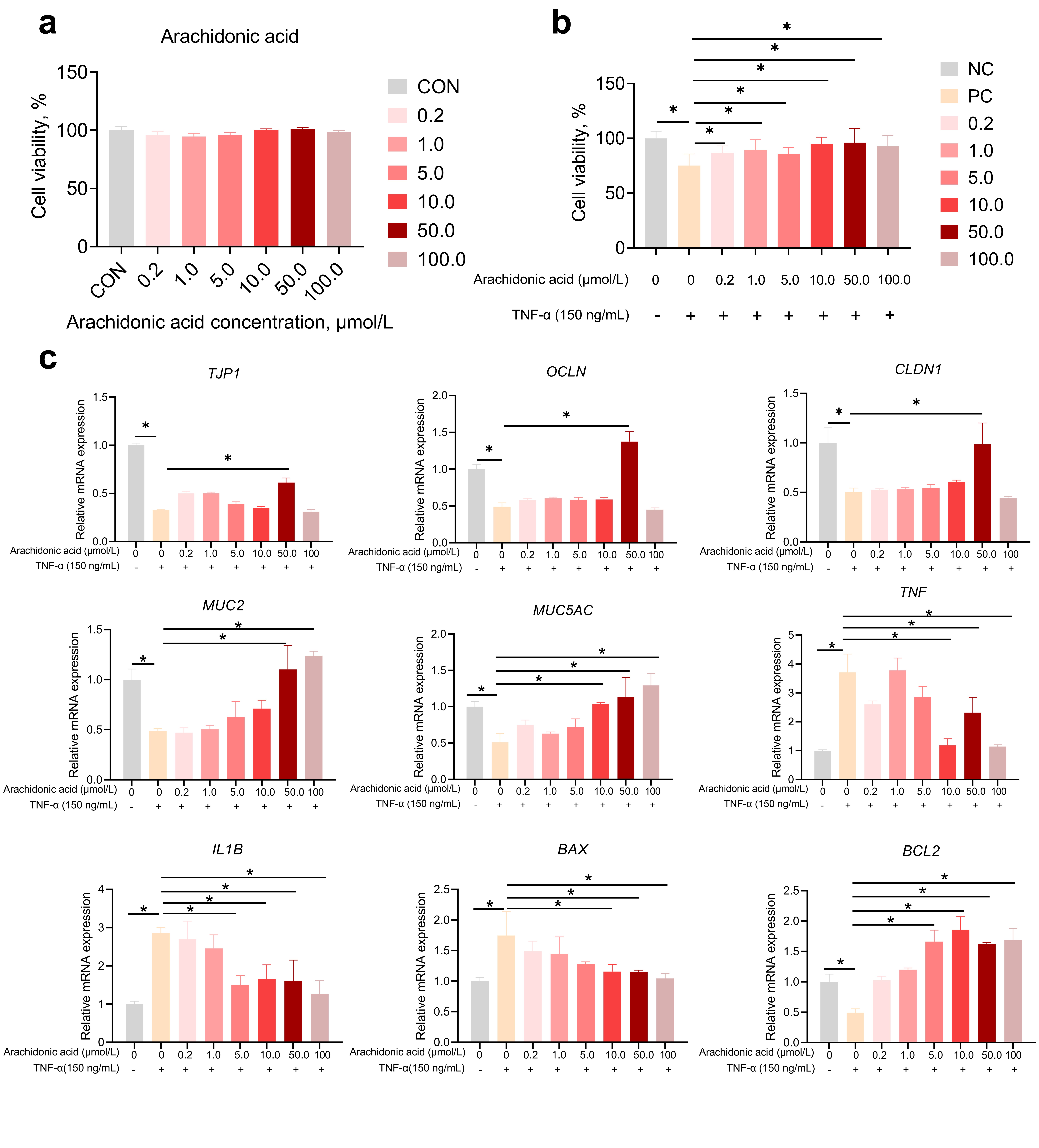


**Fig. S4** Effects of different dosage of arachidonic acid treatment on the mRNA level of tight junction protein, inflammatory factor, cell apoptosis-related gene of cells treated with TNF-α. **a** The impact of different concentrations of arachidonic acid on the viability of Caco-2 cells after 24 h. **b** The effect of different concentrations of arachidonic acid on TNF-α-induced viability of Caco-2 cells. NC: negative control, PC: positive control. **c** The effect of different concentrations of arachidonic acid on the relative expression levels of genes related to intestinal barrier, inflammation, and apoptosis in TNF-α-induced Caco-2 cells. ^*^*P* < 0.05. The biological replicates is *n* = 4, obtained from two independent experiments using different cell passages
